# Top associated SNPs in prostate cancer are significantly enriched in *cis*-expression quantitative trait loci and at transcription factor binding sites

**DOI:** 10.18632/oncotarget.2179

**Published:** 2014-07-09

**Authors:** Junfeng Jiang, Peilin Jia, Bairong Shen, Zhongming Zhao

**Affiliations:** ^1^ Department of Biomedical Informatics, Vanderbilt University School of Medicine, Nashville, TN, USA; ^2^ Center for Systems Biology, Soochow University, Suzhou, Jiangsu, China; ^3^ Center for Quantitative Sciences, Vanderbilt University, Nashville, TN, USA; ^4^ Department of Cancer Biology, Vanderbilt University School of Medicine, Nashville, TN, USA; ^5^ Department of Psychiatry, Vanderbilt University School of Medicine, Nashville, TN, USA

**Keywords:** prostate cancer, genome-wide association studies, eQTL, TFBS, regulatory variants

## Abstract

While genome-wide association studies (GWAS) have revealed thousands of disease risk single nucleotide polymorphisms (SNPs), their functions remain largely unknown. Recent studies have suggested the regulatory roles of GWAS risk variants in several common diseases; however, the complex regulatory structure in prostate cancer is unclear.

We investigated the potential regulatory roles of risk variants in two prostate cancer GWAS datasets by their interactions with expression quantitative trait loci (eQTL) and/or transcription factor binding sites (TFBSs) in three populations.

Our results indicated that the moderately associated GWAS SNPs were significantly enriched with *cis*-eQTLs and TFBSs in Caucasians (CEU), but not in African Americans (AA) or Japanese (JPT); this was also observed in an independent pan-cancer related SNPs from the GWAS Catalog. We found that the eQTL enrichment in the CEU population was tissue-specific to eQTLs from CEU lymphoblastoid cell lines. Importantly, we pinpointed two SNPs, rs2861405 and rs4766642, by overlapping results from cis-eQTL and TFBS as applied to the CEU data.

These results suggested that prostate cancer associated SNPs and pan-cancer associated SNPs are likely to play regulatory roles in CEU. However, the negative enrichment results in AA or JPT and the potential mechanisms remain to be elucidated in additional samples.

## INTRODUCTION

Prostate cancer (PrCa) is the most prevalent non-cutaneous cancer diagnosed in men in the United States, with about one in six men developing PrCa during their lifetime [1]. The genetic inflence in PrCa was estimated to be as high as 42-57% [2, 3]. Great efforts have been made during the past several decades to elucidate the underlying etiology of this disease. Among these efforts, genome-wide association studies (GWAS) have been one of the most valuable approaches to discover potential genetic susceptibilities. As of December 4, 2012, a total of 22 PrCa GWA studies have been deposited into the GWAS Catalog at the National Human Genome Research Institute (NHGRI) [4], yielding more than 100 common single nucleotide polymorphisms (SNPs) that potentially contribute to PrCa risk. However, the reported SNPs could only explain a small proportion of the genetic variances that might contribute to this disease and most significantly associated SNPs are located in non-coding regions with unknown functional annotations [4]. Furthermore, the original GWA studies typically reported only a few SNPs that reach the strigent genome-wide significance (i.e., *p* < 5×10^−8^), while neglecting those SNPs with moderate or weak significance (5×10^−8^ < *p* < 0.05).

Considering that a majority of disease-associated SNPs are located in non-coding regions that have unexplained functions, a paradigm has emerged to link associated SNPs discovered in GWAS with regulatory data, such as expression quantitative trait loci (eQTL) [5-11]. For example, Nicolae *et al*. [12] examined trait-associated SNPs collected from the NHGRI GWAS Catalog [4] and the Wellcome Trust Case Control Consortium (WTCCC) GWAS data (Crohn's disease, type 1 diabetes, and rheumatoid arthritis) and reported that trait-associated SNPs are more likely to be eQTLs. Additionally, several studies on neuropsychiatric disorders, such as schizophrenia [13, 14], bipolar disorder [15], Tourette's syndrome [16], obsessive-compulsive disorder [17], and Autism [18], displayed a similar trend that top trait-associated SNPs are more likely to be enriched with regulatory variants in eQTLs or methylation quantitative trait loci (mQTLs). Comparative studies have not been conducted on any single type of cancer yet, though there were numerous cancer GWA studies published recently. It would be interesting to examine whether cancer-associated SNPs function through their regulatory roles in a way that is similar to those in psychiatric diseases. In addition, the recent release of the data from the Encyclopedia of DNA Elements (ENCODE) project provides valuable and comprehensive annotations regarding regulatory variants in the human genome, especially transcription factor binding site (TFBS) data [19, 20].

**Figure 1:**
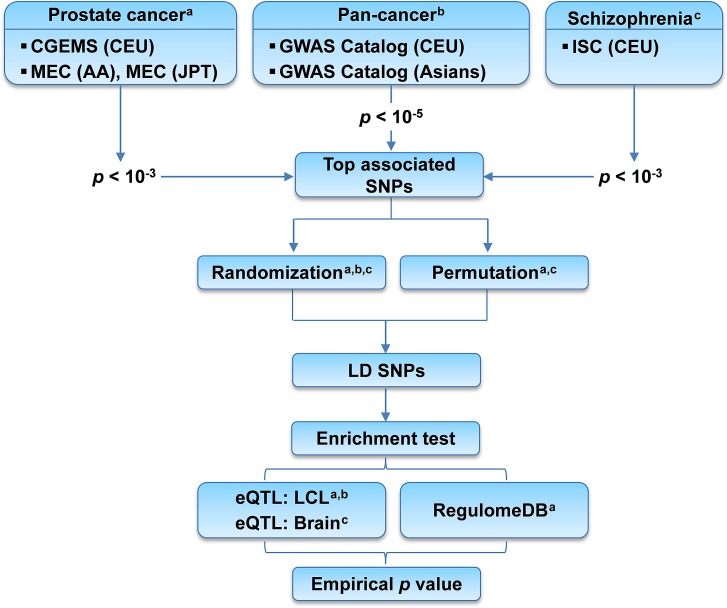
Flow chart of the enrichment analysis of top associated SNPs with prostate cancer CGEMS: Cancer Genetic Markers of Susceptibility GWAS. MEC: Multiethnic Cohort GWAS. ISC: International Schizophrenia Consortium GWAS. CEU: Caucasians. AA: African Americans. JPT: Japanese. Top associated SNPs: SNPs whose association p-values surpassed the pre-defined cutoff. LD SNPs: SNPs located in the linkage disequilibrium (LD) blocks of the top associated SNPs. LCL: lymphoblastoid cell lines.

In this work, we explored top PrCa-associated SNPs for regulatory roles in eQTLs and TFBSs. Specifically, we used two PrCa GWAS datasets: the Cancer Genetic Markers of Susceptibility (CGEMS) [21] and the Multiethnic Cohort (MEC) [22]. Considering that eQTL information relies on specific population, we examined the regulatory roles in three human populations, respectively: Caucasian (CEU), African American (AA), and Japanese (JPT) populations (Figure 1). An enrichment test was performed based on randomization and/or permutation process, whichever was applicable. We incorporated TFBS data as complementary regulation mechanisms. Our observations were further validated by using pan-cancer association SNPs collected from the GWAS Catalog [4]. We further evaluated the enrichment pattern using tissue- and/or population-matched eQTL data in top PrCa-associated SNPs. To the best of our knowledge, this is the first study that investigates the enrichment patterns of eQTL and TFBS in PrCa or any type of cancer. We observed a significant enrichment in the PrCa CEU population. Interestingly, our joint analysis of associated SNPs with eQTL and TFBS data further highlighted two SNPs, rs2861405 and rs4766642, in strong linkage disequilibrium (LD) with the PrCa-associated SNPs. These two SNPs were predicted to affect the expression of their downstream genes, i.e., *ZNF791* (by regulation through eQTL) and *CREBBP* (by regulation through TFBS) for rs2861405, and *GLTP* (eQTL) and *SPI1* (TFBS) for rs4766642. Our finding warrants future investigation of these SNPs' functions in PrCa.

## RESULTS

### Enrichment analysis with eQTL

We obtained 678, 1216, and 326 top PrCa-associated SNPs (*p* < 10^−3^) in CGEMS-CEU, MEC-AA, and MEC-JPT, respectively. As a comparison, we re-analyzed the International Schizophrenia Consortium (ISC)-CEU GWAS data, which had been demonstrated previously as a significant enrichment of eQTLs in brain data [13]. We included this data for the purpose of validating our methods as well as to compare the effect of eQTL on different diseases. We obtained 1470 schizophrenia-associated SNPs with *p* < 10^−3^.

**Figure 2:**
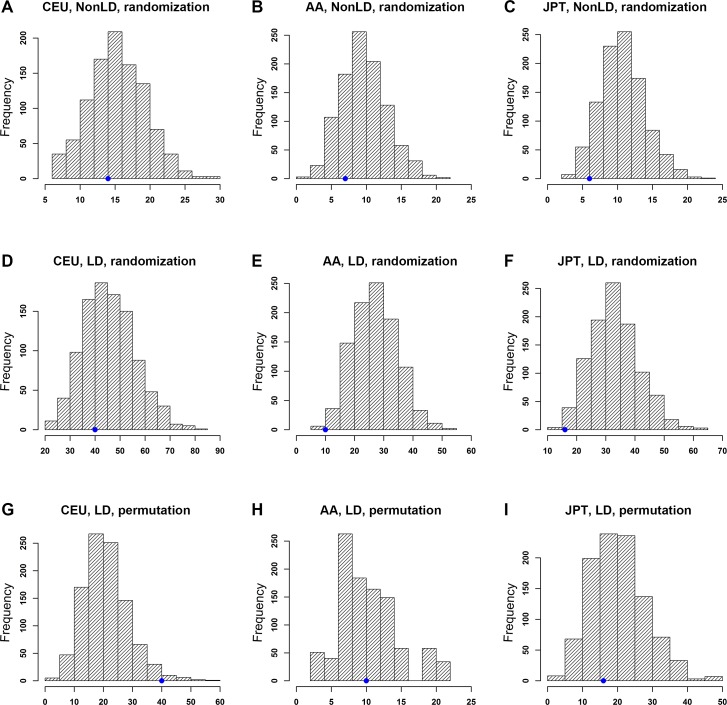
Enrichment analysis of PrCa-associated SNPs with *cis*-expression quantitative trait loci (eQTLs) X-axis: eSNP block count. Y-axis: frequency of eSNP blocks. The blue dot on each plot indicates the observed number of eSNP blocks. Note that the scales of those plots are different. Distributions of eSNP blocks using a randomization test without considering LD SNPs are shown in (A) CEU, (B) AA, and (C) JPT populations. Distributions using a randomization test considering LD SNPs are shown in (D) CEU, (E) AA, and (F) JPT populations. Distributions using a permutation test considering LD SNPs are shown in (G) CEU, (H) AA, and (I) JPT populations.

We first examined whether top PrCa-associated SNPs were enriched with lymphoblastoid cell lines (LCL) eQTLs in each population. Both the randomization and permutation tests were performed (see Material and methods). Notably, the number of *trans*-eQTLs blocks in the original GWAS dataset for the three populations was less than 3 and sometimes the number was 0. This indicates that *trans*-eQTLs in these datasets may not hava a confident estimation of the significance in the test. Thus, we only focused on *cis*-eQTLs. As shown in Figure 2, the randomization test did not indicate a significant enrichment in any of the three populations regardless of the LD SNPs being taken into consideration. Specifically, the results not considering LD SNPs were *p*_CEU_ = 0.720, *p*_AA_ = 0.867 and *p*_JPT_ = 0.979 (Figure 2A-C); and considering LD SNPs were *p*_CEU_ = 0.726, *p*_AA_ = 0.996 and *p*_JPT_ = 0.996 (Figure 2D-F, Supplementary Table 2). By applying the permutation tests, as shown in Figure 2G-I, we identified 40, 10, and 16 independent eSNP blocks in CGEMS-CEU, MEC-AA, and MEC-JPT, respectively, while the expected numbers of eSNP blocks were 21.48 (s.d. = 7.68), 10.76 (s.d. = 4.30), and 20.72 (s.d. = 8.10), respectively (Supplementary Table 2). Here, s.d. denotes standard deviation. The empirical *p* values of the permutation tests were *p*_CEU_ = 0.019, *p*_AA_ = 0.463, and *p*_JPT_ = 0.653 (Supplementary Table 2). The results above indicated that the associated SNPs in CGEMS-CEU were significantly enriched with eSNPs from LCL *cis*-eQTL data but were not enriched in either the MEC-AA or MEC-JPT population.

As described in the Materials and Methods, the randomization test tends to overestimate the number of expected independent LD blocks because it ignores the LD structure across the genome. Thus, the generated null distribution would likely be inflated with eSNPs and lead to a false negative discovery. On the contrary, the permutation test is expected to be more accurate in identifying regulatory information than the randomization test, though it requires raw genotyping data and is computationally time consuming. To confirm this hypothesis, we applied both randomization and permutation tests to the ISC-CEU data, which has previously shown that trait-associated SNPs in this dataset were significantly enriched with brain eQTL through the randomization test [13]. Our randomization test indeed revealed a pattern that is similar to what was observed in the previous study (Supplementary Figure 1A, B). The permutation test showed a much stronger (Supplementary Figure 1C) enrichment pattern than the randomization test, indicating that the latter might overestimate the expected number of eSNPs (or eSNP blocks). As shown in Table 1, for GWAS data that contains a large proportion of eSNPs among top associated SNPs (e.g., ISC-CEU), we observed a significant enrichment pattern in both randomization and permutation tests (Supplementary Figure 1); however, for GWAS data with a smaller proportion of eSNPs, such as PrCa GWAS, overestimation of eSNPs may lead to a false negative discovery resulting from the randomization test. In both cases, the permutation test seems to have better power to estimate a null distribution reflecting the true association. Therefore, in the following analyses, we applied a permutation approach to evaluate the significance of enrichment, as long as the genotyping data was available.

**Table 1:** Proportion of expression quantitative trait loci (eQTL) SNPs under different *p*-value cutoffs in prostate cancer (PrCa) and schizophrenia (SCZ) GWAS Abbreviations: CGEMS: Cancer Genetic Markers of Susceptibility. CEU: Caucasians. MEC: Multiethnic Cohort. AA: African Americans. JPT: Japanese. ISC: International Schizophrenia Consortium.

Disease	GWAS dataset	Population	*p*-value cutoff									
			1×10^−6^	5×10^−6^	1×10^−5^	5×10^−5^	1×10^−4^	5×10^−4^	1×10^−3^	0.01	0.1	1
PrCa	CGEMS	CEU	0	0	0	0	0.010	0.013	0.022	0.027	0.024	0.024
PrCa	MEC	AA	0	0	0	0	0	0.005	0.007	0.006	0.008	0.008
PrCa	MEC	JPT	0	0	0	0	0	0.014	0.028	0.037	0.035	0.033
SCZ	ISC	CEU	0.250	0.143	0.137	0.137	0.110	0.073	0.048	0.013	0.007	0.004

### Enrichment analysis with transcription factor binding sites

**Figure 3:**
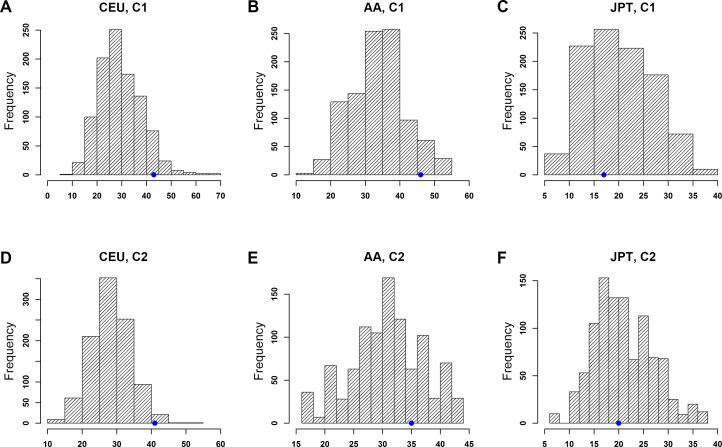
Enrichment analysis of top PrCa-associated SNPs with transcription factor binding sites (TFBSs) of RegulomeDB Category 1 (C1) and Category 2 (C2) using the permutation test X-axis: tSNP block count. Y-axis: frequency of tSNP blocks. The blue dot on each plot indicates the observed number of tSNP blocks. Note that the scales of those plots are different. Distributions of tSNPs blocks considering LD SNPs are shown in (A) CEU, C1, (B) AA, C1, (C) JPT, C1, (D) CEU, C2, (E) AA, C2, and (F) JPT, C2.

Similar to eQTL enrichment analyses, we examined whether top PrCa-associated SNPs were enriched with tSNPs, i.e. those SNPs in TFBSs retrieved from C1 and C2 categories data of RegulomeDB. As shown in Figure 3, the top PrCa-associated SNPs in CGEMS-CEU were significantly enriched with C2 SNPs (*p*_CEU_ = 0.014, Figure 3D, Supplementary Table 2), while this significance was slightly above the *p* < 0.05 threshold when using C1 data (*p*_CEU_ = 0.068, Figure 3A, Supplementary Table 2). We did not observe any significant enrichment in either the MEC-AA or MEC-JPT population in data either from C1 (*p*_AA_ = 0.061, Figure 3B; *p*_JPT_ = 0.603, Figure 3C) or C2 (*p*_AA_ = 0.230, Figure 3E, *p*_JPT_ = 0.502, Figure 3F). When we examined the pattern in each subcategory, we found that most signals (94.9% from the observed data and 88.0% (s.d. = 4.1%) from the 1000 permutation sets) in C1 fell into subgroup 1f, which includes eQTL and minimal TF binding/DNase peak evidence [20]. This analysis partially repeated our eQTL enrichment result above but using mixed population of eQTL data, providing confidence in eQTL regulation of the top PrCa-associated SNPs in CGEMS-CEU.

### Replication of eQTL and TFBS enrichment using GWAS Catalog SNPs

**Figure 4:**
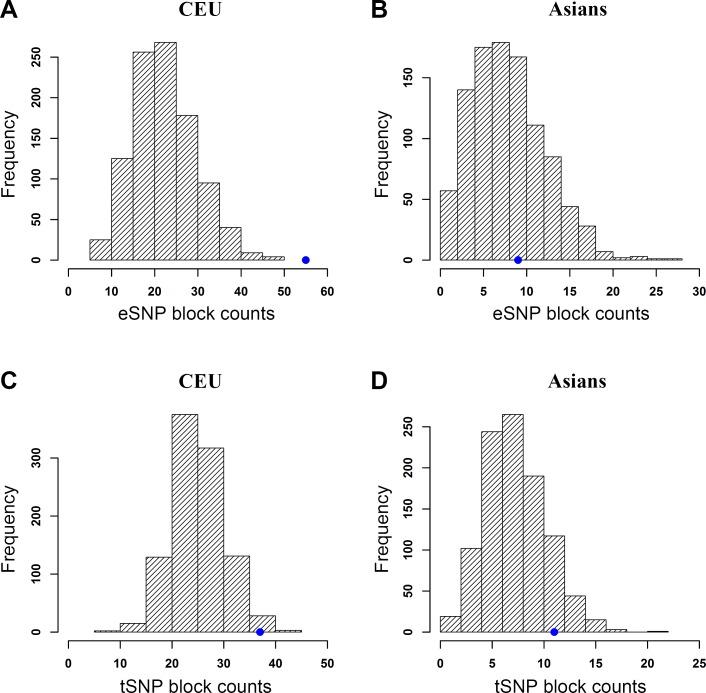
Enrichment analysis of cancer-associated SNPs with *cis*-expression quantitative trait loci (eQTLs) and transcription factor binding sites (TFBSs) of RegulomeDB Category 2 (C2) using a randomization test The blue dot on each plot indicates the observed number of eSNP/tSNP blocks. Note that the scales of those plots are different. Distributions of eSNP/tSNP blocks considering LD SNPs are shown in (A) CEU, cis-eQTL, (B) Asians, cis-eQTL, (C) CEU, C2, and (D) Asians, C2.

We collected 254 cancer-associated SNPs from 64 studies of 14 different types of cancer in CEU (Supplementary Table 3). Data for the AA population was not sufficient because only 26 SNPs were deposited in the GWAS Catalog. Considering the small number of cancer-associated SNPs for the JPT population in the GWAS Catalog, we collected 79 cancer-associated SNPs from 19 studies covering 8 different types of cancer in the Asian population (Supplementary Table 4). As shown in Figure 4A and 4C, we found that the cancer-associated SNPs were significantly enriched with *cis*-eQTLs (*p* < 0.001) and TFBSs (*p* = 0.021) in the CEU population (Supplementary Table 2). For the cancer-associated SNPs in the Asian population, we did not observe such a significant enrichment with either *cis*-eQTLs (Figure 4B) or TFBSs (Figure 4D). Next, we examined the enrichment of prostate cancer GWAS Catalog SNPs with eQTLs and TFBSs. We found 99 and 15 GWAS Catalog SNPs for PrCa in the CEU and Asian populations, respectively. For CEU, the PrCa GWAS Catalog SNPs showed similar enrichment significance with eQTLs (*p* = 0.012, 21 eSNP blocks) to CGEMS-CEU GWAS data (*p* = 0.019, 40 eSNP blocks), but not with TFBSs (*p* = 0.298, 12 tSNP blocks, compared to *p* = 0.014, 41 tSNP blocks in CGEMS-CEU GWAS data) (Supplementary Table 2). We noted that the number of tSNP blocks (12) based on PrCa CEU GWAS Catalog SNPs might be too small to have a reliable statistical test. Overall, the analysis of GWAS Catalog SNPs indicates that the top associated SNPs are likely to function through regulatory roles (e.g., eQTLs) in CEU PrCa samples. For the PrCa SNPs in Asian population, the number of eSNP/tSNP blocks was 0 and 1, respectively, in all random SNP sets. These numbers are too small to perform a qualified enrichment test. Thus, we did not perform the enrichment test in Asian population.

Due to the lack of appropriate genotyping data, we could not perform the permutation test. As we stated above, the bias in the randomization test leans toward false negative results only. Therefore, the observed positive enrichment pattern in CEU suggests that top cancer-associated SNPs are more likely to function through regulatory roles, i.e., via eQTLs or TFBSs, in CEU PrCa samples. Caution should be taken, however, when a similar pattern is not present in the Asian or other population. Further investigation with more data will help us to better understand this regulatory system among PrCa populations.

### Specificities of eQTL enrichment

The above analyses were conducted using LCL *cis*-eQTL data in the matched population for PrCa-associated SNPs. We further asked whether the enrichment pattern we observed in CGEMS-CEU is conserved among eQTLs with different tissues or different populations. The results were shown in Table 2. First, our results showed that the top PrCa-associated SNPs were significantly enriched with LCL eQTLs but not with brain eQTLs (*p* = 0.211) or liver eQTLs (*p* = 0.196), suggesting that the enrichment pattern in CEU might be tissue-specific. Second, the top PrCa-associated SNPs in CEU did not show significant signals that were enriched with eQTLs derived from AA (*p* = 0.173) or JPT (*p* = 0.063), further highlighting the necessity to use the population-matched eQTL data for GWAS data analysis.

**Table 2:** Summary of expression quantitative trait loci (eQTL) enrichment under different scenarios using Cancer Genetic Markers of Susceptibility (CGEMS) prostate cancer GWAS data Abbreviations: eSNP: eQTL SNP. CEU: Caucasians. LCL: lymphoblastoid cell lines. AA: African Americans. JPT: Japanese.

	# observed eSNP blocks	# expected eSNP blocks (s.d.)	*p*-value
CEU, *cis*-eQTL, LCL	40	21.48 (7.68)	0.019
Tissue specificity			
CEU, *cis*-eQTL, brain	4	3.00 (2.37)	0.211
CEU, *cis*-eQTL, liver	3	2.23 (1.52)	0.196
eQTL population specificity			
AA, *cis*-eQTL, LCL	10	6.71 (3.28)	0.173
JPT, *cis*-eQTL, LCL	40	26.87 (8.21)	0.063
CEU+AA+JPT, cis-eQTL, LCL	63	36.90 (10.16)	0.011

### Combining cis-eQTL and TFBS for better detection of candidate susceptibility loci

We further checked the results from *cis*-eQTL and TFBS enrichment analyses among all LD SNPs of CGEMS-CEU. Among the identified 131 *cis*-eSNPs, two were found to be located in the TFBSs: rs4766642 and rs2861405. These two SNPs were not directly genotyped in the CGEMS-CEU GWAS. Rather, they were located in strong LD with the SNPs that were genotyped: rs4766642 is in strong LD with the genotyped SNP rs10850830 (*r*^2^ = 0.95; *p*_GWAS_ = 9.17×10^−4^), while rs2861405 is in strong LD with the genotyped SNPs rs4804202 (*r*^2^ = 1.00; *p*_GWAS_ = 2.63×10^−4^) and rs8107642 (*r*^2^ = 0.91; *p*_GWAS_ = 1.89×10^−4^). Because these two SNPs, rs4766642 and rs2861405, are both eSNPs and tSNPs, they provided candidates for future investigation.

## DISCUSSION

We performed a comprehensive investigation of top PrCa-associated SNPs for their potential roles in regulating gene expression through eQTL and/or TFBS. We attempted to study the regulatory roles for two types of association data: moderately significant SNPs that were associated with PrCa and cancer-associated SNPs from the GWAS Catalog that reached the genome-wide significance level. To our knowledge, this is the first investigation of the enrichment of associated SNPs with eQTLs and TFBS in prostate cancer in different populations.

Our results not only revealed the potential regulatory mechanisms of the top PrCa-associated SNPs, but also highlighted two candidate SNPs that might play important roles in the disease. Notably, in the original CGEMS study [21], no SNP was able to reach the genome-wide significance level (*p* < 5×10^−8^). Through the examination of both eQTL and TFBS data, we identified two regulatory SNPs, rs2861405 and rs4766642, which were not directly genotyped in the GWAS data yet were shown in strong LD with the top associated SNPs. Both of the target genes that were regulated by these two SNPs have been previously reported involving in PrCa, thus, at least to some extent, proving the integrative analysis of eQTL and TFBS might increase the ability to detect true association signals in prostate cancer and other complex diseases. Specifically, *ZNF791*, whose expression is regulated by rs2861405 through eQTL, encodes a member of the zinc finger protein family that have been reported as associated with prostate cancer at gene expression and protein levels [23, 24]. SNP rs2861405 is located at the TFBS of *CREBBP*, a gene often considered to be a PrCa biomarker [25, 26]. This gene plays critical roles in the prostate cancer pathway (KEGG ID: has05215) [27]. The other SNP, rs4766642, reportedly regulates the expression of *GLTP* through eQTL in prostate cancer cells, which could have an important contribution to the regulation of endothelial cell mobility [28]. Moreover, these two SNPs were also mapped in the DNase hypersensitive site of the ENCODE prostate cancer cell line, LNCaP, further supporting their roles in regulating gene expression [19, 20].

This study raised several methodology issues that may complicate the analysis of disease-associated SNPs with eQTL/TFBS data and, thus, provided a reference for similar analyses in future work. First, due to LD structures, the widely used randomization test may result in false negative findings when the enrichment is not strong. Alternatively, the permutation test is robust, but it also requires genotype data and is computationally intensive. Second, the incorporation of LD structure information is important for the discovery of regulatory patterns, especially when the GWA studies and the eQTL studies are conducted on different array platforms. For example, in our study, we observed 26 more eSNP blocks after considering LD SNPs (Supplementary Table 2). Third, a number of confounding factors may influence the comparison of GWAS data with eQTL data, including tissue specificity and population structure (Table 2).

This study has the following limitations, which could be improved in future investigations. First, there has been no eQTL data profiled in prostate tissue; the eQTL data currently available is mainly from LCL, brain, and liver tissues. Our observations were based on LCL eQTL data, the closest tissue we could find for prostate cancer. Prostate tissue-specific eQTL data will likely be generated in the near future, such as from the Genotype-Tissue Expression (GTEx) project. Future studies that utilize the genetic information from disease-specific (i.e., prostate tissue) will make the conclusions solid. Second, the lack of significance in the AA or JPT population in this study is inconclusive and requires replication in future work, as the existing prostate cancer GWAS data and eQTL/TFBS data is currently limited. For example, the amount of eQTL data available for the AA population (# eQTLs = 13,995) is only ~33.1% of that in the CEU population (# eQTLs = 42,301), which reduced the reliability of our observations in the AA data. Notably, the samples used for detection of eQTL in AA were comparable to those in CEU, indicating that AA samples tend to have fewer eQTLs regardless of sample size [33]. As for the JPT population, though the eQTL data is sufficient for our analysis, the sample size in the MEC-JPT GWAS dataset was only 392 (158 cases and 234 controls), which may not have sufficient power to detect PrCa-associated SNPs in the JPT population. Third, the genotyping platforms used in eQTL studies and in GWA studies are often different, which introduces difficulties in forming direct comparisons between eSNPs and disease-associated SNPs. In our work, we employed the LD expansion strategy while, ideally, imputation should be a robust way to eliminate inconsistency among platforms. Due to the heavy computational load, we did not perform imputation on the GWAS data but will include it in our future work.

In summary, we conducted comprehensive enrichment analyses of the top associated SNPs in eQTLs and TFBSs in three populations (CEU, AA, and JPT) from two PrCa GWAS datasets, CGEMS and MEC. Our results supported the hypothesis that prostate cancer risk SNPs in the CEU population may act through *cis*-regulators in the expression of their target genes, which has not been observed in the AA or JPT population yet. Our preliminary work also revealed that the pattern might be specific to eQTL data in the matched disease-relevant tissue and population. We identified two promising regulartory SNPs (rs2861405 and rs4766642) in PrCa. Our work provides insights and guidance, both biologically and methodologically, for future investigations of the regulatory system of prostate cancer and other complex diseases.

## MATERIAL AND METHODS

### Genotype datasets

The CGEMS prostate cancer GWAS [21] dataset was generated using Illumina HumanHap300 (Phase 1A) and Illumina HumanHap240 (Phase 1B) arrays, resulting in approximately 550,000 SNPs for 1172 prostate cancer patients and 1157 controls of European ancestry from the Prostate, Lung, Colon and Ovarian (PLCO) Cancer Screening Trial. Data was downloaded from the National Center for Biotechnology Information (NCBI) dbGaP with approved access (request: # 5662-1). Following our previous study [29], we obtained a total of 506,216 SNPs from 2243 samples, and denoted the data hereafter as CGEMS-CEU.

The MEC GWAS were conducted by genotyping using the Illumina Human1M_Duov3_B array or the Human660W_Quad_v1_A array. The samples were collected in men of AA, JPT, and Latino (LTN) populations [22]. We only used samples from AA and JPT, and denoted them as MEC-AA and MEC-JPT, since eQTL data has been very limited so far for the LTN population. For AA samples, we collected 996,050 SNPs genotyped in 1371 cases and 1313 controls using Illumina Human1M_Duov3_B. For JPT samples, we collected 458,616 SNPs genotyped in 158 cases and 234 controls using Human660W_Quad_v1_A. The association test was conducted following the previous study [22].

The schizophrenia GWAS dataset was from the International Schizophrenia Consortium (ISC). We denoted the data hereafter as ISC-CEU. A detailed description can be found in previous studies [30, 31].

HapMap genotype data (release 27, including samples from phase I, II, and III) were downloaded from the HapMap website [32]. The LD data of HapMap samples was downloaded for the CEU, AA, and JPT, respectively.

### eQTL and TFBS datasets

We utilized human eQTL association data from a recently developed public database, seeQTL [33], which collected 9 unrelated HapMap studies of lymphoblastoid cell lines [6, 7, 9-11, 34, 35], human cortical samples [5], and monocytes [36]. In the seeQTL database, eQTL data from these previous studies was collected and re-analyzed using a combination of quality control, population stratification, and false discovery rate (FDR) assessement to generate *cis*- and *trans*-eQTLs. In our analysis, as shown in Supplementary Table 1, we used the LCL and brain eQTL data by significance (q-value) < 0.2 (default). Here, q-values were obtained by adjusting regression *p*-values using the Bejaminin-Hochberg correction method [37], as described in [33]. We also incorporated liver eQTL data reported by Innocenti *et al*. [38], which was retrieved from the eQTL Browser database (http://eqtl.uchicago.edu/help.html).

RegulomeDB [20] is a comprehensive resource for regulatory variants in the human genome, primarily based on the ENCODE data [19], and other resources, such as ChIP-seq data from the NCBI Sequence Read Archive (SRA) and eQTL data from recent publications. Of note, the data collected by RegulomeDB is not specifically distinguished by population or tissue type. RegulomeDB has six categories of functional SNPs with systematic ranking scores. SNPs in C1 mainly contain eQTL and binding affinity signals, with 6 subcategories from 1a to 1f that further classify SNPs by decreasing confidence. SNPs in C2 are annotated as “likely to affect binding.” The other four categories (categories 4-6) represent weak or minimal binding evidence for the functional SNPs [20]. Correspondingly, we only considered category 1 (C1) and category 2 (C2) in our analysis for the enrichment test, which is shown in Supplementary Table 1.

### Enrichment tests and evaluation

Due to the lack of significantly associated SNPs surpassing genome-wide significance (*p* < 5×10^−8^) in either of the two original GWAS datasets, we denoted the top PrCa-associated SNPs as those with moderate significance (e.g., *p* < 10^−3^) [15]. In this study, we applied two statistical approaches, the randomization and the permutation tests, to build the null distribution of simulated SNPs at random cases for an enrichment test of eQTLs and/or TFBSs. Throughout this work, all enrichment tests were performed in a population-specific way, e.g., significant GWAS SNPs obtained in the CEU population were tested in the corresponding CEU eQTL or TFBS data, unless otherwise specified.

Following the work as described in Nicolae *et al*. [12], we classified the SNPs to the 10 MAF bins, which were constructed with an interval of 5%, i.e., 0-5%, 5-10%, …, 45-50%. We generated 1000 random SNP sets, in which the same number of disease-associated SNPs with the same distribution of MAF bins as in the actual GWAS dataset was randomly sampled from all the SNPs genotyped on the GWAS platform without replacement. These random SNPs were then mapped to SNPs of eQTLs or located at the TFBSs (hereafter denoted as eSNPs and tSNPs, respectively) to form a null distribution to assess the significance.

Although the randomization test has been widely applied in many diseases [13, 15, 16], one recognized disadvantage is the ignorance of LD structures among SNPs, which may complicate the results and lead to false negative findings in practical cases. In contrast, in a randomization test, the randomly selected SNPs from the genome are more likely to be independent because the randomization process essentially disregards the LD structure. Due to this potential bias, we proposed counting the number of LD blocks instead of using the raw number of eSNPs to estimate the significance level. We define an LD block as a cluster of SNPs that are located in a genomic region in which any two of the SNPs have *r^2^* ≥ 0.5, where *r^2^* is the squared LD correlation coefficient. We used PLINK [39] to calculate the independent blocks of eSNPs and tSNPs. Accordingly, the empirical *p* value of the randomization test is defined as 
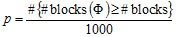
, where Φ denotes a randomization dataset.

Alternatively, the permutation test generates random datasets by randomly swapping cases and controls while keeping the same number of cases and controls in the population. In this way, the LD structure within individuals remains intact. We generated 1000 sets of phenotype files and conducted an association test using the same statistical strategy [29]. The top associated SNPs in each permutation dataset is similarly defined using the same threshold (e.g., *p* < 10^−3^), and the number of blocks is recorded to compute an empirical *p* value: 
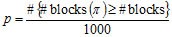
, where π denotes a permutation dataset.

For both the randomization and permutation tests, we further extracted “LD SNPs,” which were defined as those with *r*^2^ ≥ 0.5 to any of the top disease-associated SNPs according to the LD data derived from the HapMap samples of the same population. Then, we applied a similar approach as described above to calculate the significance level.

As a replication, we examined all the top cancer-associated SNPs deposited in the NHGRI GWAS Catalog [4]. Here, we denoted the top cancer-associated SNPs as those surpassing the genome-wide significance level (*p* < 10^−5^) [12]. To collect cancer-associated SNPs, we manually extracted the SNPs deposited in the GWAS Catalog specifically for the European and Asian populations (samples in the AA population were neglected because only two GWA studies were reported) (as of December 4, 2012, http://www.genome.gov/gwastudies/). Since the raw genotype data of these GWA studies are mostly unavailable, we only performed a randomization test. We followed the same procedure as described above; however, we used the combined SNPs from Affymetrix Genome-Wide Human SNP Array 6.0 and Illumina's High Density Human 1M-Duo as the genotyped SNPs on the GWAS platforms. Cancer-associated SNPs that did not have MAF information in the combined platform were excluded.

## SUPPLIMENTARY MATERIAL FIGURE AND TABLES


