# Supplementary material for: Top associated SNPs in prostate cancer are significantly enriched in cis-expression quantitative trait loci and at transcription factor binding sites
Source: Oncotarget. 2014 Jul 9;5(15):6168–77. doi: 10.18632/oncotarget.2179 (PMC4171620; doi:10.18632/oncotarget.2179)
Supplement: Supplementary file 1 [file oncotarget-05-6168-s001.pdf]

Top associated SNPs in prostate cancer are significantly enriched in *cis*-expression quantitative trait loci and at transcription factor binding sites

Supplimentary Material

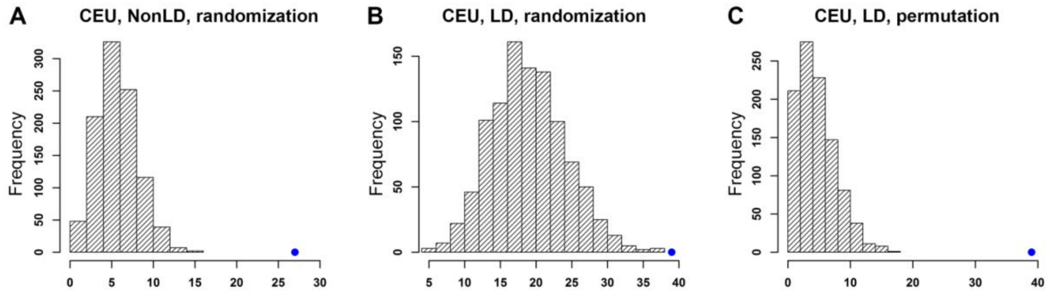

Supplementary Figure 1: Enrichment analysis of top associated SNPs in ISC-CEU GWAS with *cis*-expression quantitative trait loci (eQTLs). X-axis: eSNP block count. Y-axis: frequency of eSNP blocks. The blue dot on each plot indicates the observed number of eSNP blocks. Note that the scales of those plots are different. Distributions using randomization test with or without considering LD SNPs are shown in (a) NonLD and (b) LD. Distribution using permutation test considering LD SNPs is shown in (c).

Supplementary Table 1: Summary of eQTL and TFBS datasets

	Population	Tissue	Database/reference
eQTL	CEU[c]	LCL[d]	SeeQTL[1]
	AA[e]	LCL	SeeQTL
	JPT[f]	LCL	SeeQTL
	CEU	brain	SeeQTL
	CEU	liver	Innocenti *et al.*[2]
	**Category**	**Description**	**Database**
TFBS	1	eQTL and binding affinity signals	RegulomeDB[3]
	2	Likely to affect binding	RegulomeDB

Abbreviations: eQTL: expression quantitative trait loci. TFBS: transcription factor binding site. CEU: Caucasians. LCL: lymphoblastoid cell lines. AA: African Americans. JPT: Japanese.

References

1 Xia K, Shabalin AA, Huang S, Madar V, Zhou YH, Wang W, Zou F, Sun W, Sullivan PF, Wright FA. seeQTL: a searchable database for human eQTLs. *Bioinformatics* 2012;**28**:451-52.

2 Innocenti F, Cooper GM, Stanaway IB, Gamazon ER, Smith JD, Mirkov S, Ramirez J, Liu W, Lin YS, Moloney C, Aldred SF, Trinklein ND, et al. Identification, replication, and functional fine-mapping of expression quantitative trait loci in primary human liver tissue. *PLoS Genet* 2011;**7**:e1002078.

3 Boyle AP, Hong EL, Hariharan M, Cheng Y, Schaub MA, Kasowski M, Karczewski KJ, Park J, Hitz BC, Weng S, Cherry JM, Snyder M. Annotation of functional variation in personal genomes using RegulomeDB. *Genome Res* 2012;**22**:1790-97.

Supplementary Table 2: Summary of enrichment with eQTLs and TFBSs for CGEMS-CEU, MEC-AA, MEC-JPT, ISC-CEU, GWAS Catalog-CEU, and GWAS Catalog-Asians

eQTL enrichment									
	Randomization (NonLD)			Randomization (LD)			Permutation (LD)		
	# observed eSNP blocks	# expected eSNP blocks (s.d.)	*p-value*	# observed eSNP blocks	# expected eSNP blocks (s.d.)	*p-value*	# observed eSNP blocks	# expected eSNP blocks (s.d.)	*p-value*
CGEMS-CEU	14	15.83 (4.03)	0.720	40	46.19 (10.34)	0.726	40	21.48 (7.68)	**0.019**
MEC-AA	7	10.15 (3.25)	0.867	10	27.50 (7.51)	0.996	10	10.76 (4.30)	0.463
MEC-JPT	6	11.24 (3.19)	0.979	16	33.56 (8.24)	0.996	16	20.72 (8.10)	0.653
ISC-CEU	27	6.18 (2.43)	**<0.001**	39	18.96 (5.19)	**<0.001**	39	5.10 (3.06)	**<0.001**
GWAS Catalog-CEU				55	22.87 (7.20)	**<0.001**			
GWAS Catalog-CEU, PrCa				21	9.02 (4.34)	0.012			
GWAS Catalog-Asians				9	8.39 (4.26)	0.371			

TFBS enrichment									
	Randomization C2 (LD)			Permutation C1 (LD)			Permutation C2 (LD)		
	# observed tSNP blocks	# expected tSNP blocks (s.d.)	*p-value*	# observed tSNP blocks	# expected tSNP blocks (s.d.)	*p-value*	# observed tSNP blocks	# expected tSNP blocks (s.d.)	*p-value*
CGEMS-CEU				43	29.96 (8.61)	0.068	41	28.68 (5.69)	**0.014**
MEC-AA				46	34.06 (8.12)	0.061	35	31.22 (6.14)	0.230
MEC-JPT				16	20.54 (6.63)	0.603	20	21.38 (5.92)	0.502
GWAS Catalog-CEU	37	25.55 (5.06)	**0.021**						
GWAS Catalog-CEU, PrCa	12	9.90(3.29)	0.298						
GWAS Catalog-Asians	11	7.78 (2.94)	0.180						

Abbreviations: eQTLs: expression quantitative trait loci. TFBSs: transcription factor binding sites. CGEMS: Cancer Genetic Markers of Susceptibility. CEU: Caucasians. MEC: Multiethnic Cohort. AA: African Americans. JPT: Japanese. ISC: International Schizophrenia Consortium. GWAS: genome-wide association studies. NonLD: without linkage disequilibrium expansion. LD: linkage disequilibrium.
eSNP: eQTL SNP. tSNP: TFBS SNP. s.d.: standard deviation.C1: RegulomeDB Category 1. C2: RegulomeDB Category 2.

Supplementary Table 3: Cancer-associated SNPs in Caucasian (CEU) population extracted from GWAS Catalog

Date added to Catalog	First Author	PubMed ID	Study	Disease/trait	SNP ID	*p*-value
2012/11/30	Siddiq A	22976474	A meta-analysis of genome-wide association studies of breast cancer identifies two novel susceptibility loci at 6q14 and 20q11.	Breast cancer	rs9383938	2.00E-10
2012/11/30	Siddiq A	22976474	A meta-analysis of genome-wide association studies of breast cancer identifies two novel susceptibility loci at 6q14 and 20q11.	Breast cancer	rs8100241	4.00E-08
2012/11/30	Siddiq A	22976474	A meta-analysis of genome-wide association studies of breast cancer identifies two novel susceptibility loci at 6q14 and 20q11.	Breast cancer	rs17530068	3.00E-07
2012/11/30	Siddiq A	22976474	A meta-analysis of genome-wide association studies of breast cancer identifies two novel susceptibility loci at 6q14 and 20q11.	Breast cancer	rs2284378	1.00E-08
2012/11/22	Timofeeva MN	22899653	Influence of common genetic variation on lung cancer risk: meta-analysis of 14 900 cases and 29 485 controls.	Lung cancer	rs10849605	6.00E-07
2012/10/9	Rajaraman P	22886559	Genome-wide association study of glioma and meta-analysis.	Glioma	rs2736100	4.00E-09
2012/10/9	Rajaraman P	22886559	Genome-wide association study of glioma and meta-analysis.	Glioma	rs6010620	1.00E-10
2012/10/9	Rajaraman P	22886559	Genome-wide association study of glioma and meta-analysis.	Glioma	rs4977756	1.00E-08
2012/7/17	Dunlop MG	22634755	Common variation near CDKN1A, POLD3 and SHROOM2 influences colorectal cancer risk.	Colorectal cancer	rs3824999	4.00E-10
2012/7/17	Dunlop MG	22634755	Common variation near CDKN1A, POLD3 and SHROOM2 influences colorectal cancer risk.	Colorectal cancer	rs5934683	7.00E-10
2012/7/17	Dunlop MG	22634755	Common variation near CDKN1A, POLD3 and SHROOM2 influences colorectal cancer risk.	Colorectal cancer	rs1321311	1.00E-10
2012/7/13	Turnbull C	22544364	A genome-wide association study identifies susceptibility loci for Wilms tumor.	Wilms tumor	rs807624	1.00E-14
2012/7/13	Turnbull C	22544364	A genome-wide association study identifies susceptibility loci for Wilms tumor.	Wilms tumor	rs5955543	1.00E-09
2012/7/13	Turnbull C	22544364	A genome-wide association study identifies susceptibility loci for Wilms tumor.	Wilms tumor	rs2283873	5.00E-12
2012/7/13	Turnbull C	22544364	A genome-wide association study identifies susceptibility loci for Wilms tumor.	Wilms tumor	rs790356	4.00E-15
2012/7/13	Turnbull C	22544364	A genome-wide association study identifies susceptibility loci for Wilms tumor.	Wilms tumor	rs1027643	5.00E-10
2012/7/13	Turnbull C	22544364	A genome-wide association study identifies susceptibility loci for Wilms tumor.	Wilms tumor	rs2495478	1.00E-07
2012/7/13	Turnbull C	22544364	A genome-wide association study identifies susceptibility loci for Wilms tumor.	Wilms tumor	rs3755132	1.00E-14
2012/5/16	Xiao Y	22472174	SSBP2 variants are associated with survival in glioblastoma patients.	Glioblastoma	rs7732320	1.00E-06
2012/2/9	Gudmundsson J	22267200	Discovery of common variants associated with low TSH levels and thyroid cancer risk.	Thyroid cancer	rs2439302	2.00E-09
2012/2/9	Gudmundsson J	22267200	Discovery of common variants associated with low TSH levels and thyroid cancer risk.	Thyroid cancer	rs966423	1.00E-09
2012/3/31	Tao S	22219177	A genome-wide search for loci interacting with known prostate cancer risk-associated genetic variants.	Prostate cancer	rs7694725	2.00E-06
2012/3/31	Tao S	22219177	A genome-wide search for loci interacting with known prostate cancer risk-	Prostate cancer	rs10086908	2.00E-06

			associated genetic variants.			
2012/3/31	Tao S	22219177	A genome-wide search for loci interacting with known prostate cancer risk-associated genetic variants.	Prostate cancer	rs1447295	2.00E-06
2012/3/31	Tao S	22219177	A genome-wide search for loci interacting with known prostate cancer risk-associated genetic variants.	Prostate cancer	rs6766510	2.00E-06
2012/3/31	Tao S	22219177	A genome-wide search for loci interacting with known prostate cancer risk-associated genetic variants.	Prostate cancer	rs735172	2.00E-06
2012/3/31	Tao S	22219177	A genome-wide search for loci interacting with known prostate cancer risk-associated genetic variants.	Prostate cancer	rs2711721	2.00E-06
2012/3/31	Tao S	22219177	A genome-wide search for loci interacting with known prostate cancer risk-associated genetic variants.	Prostate cancer	rs10993994	2.00E-06
2012/3/31	Tao S	22219177	A genome-wide search for loci interacting with known prostate cancer risk-associated genetic variants.	Prostate cancer	rs887391	2.00E-06
2012/3/31	Tao S	22219177	A genome-wide search for loci interacting with known prostate cancer risk-associated genetic variants.	Prostate cancer	rs5759167	2.00E-06
2012/3/31	Tao S	22219177	A genome-wide search for loci interacting with known prostate cancer risk-associated genetic variants.	Prostate cancer	rs4463179	2.00E-06
2012/3/31	Tao S	22219177	A genome-wide search for loci interacting with known prostate cancer risk-associated genetic variants.	Prostate cancer	rs10934853	3.00E-06
2012/3/31	Tao S	22219177	A genome-wide search for loci interacting with known prostate cancer risk-associated genetic variants.	Prostate cancer	rs2400997	3.00E-06
2012/3/31	Tao S	22219177	A genome-wide search for loci interacting with known prostate cancer risk-associated genetic variants.	Prostate cancer	rs6465657	3.00E-06
2012/3/31	Tao S	22219177	A genome-wide search for loci interacting with known prostate cancer risk-associated genetic variants.	Prostate cancer	rs2219968	6.00E-07
2012/3/31	Tao S	22219177	A genome-wide search for loci interacting with known prostate cancer risk-associated genetic variants.	Prostate cancer	rs10795917	7.00E-07
2012/3/31	Tao S	22219177	A genome-wide search for loci interacting with known prostate cancer risk-associated genetic variants.	Prostate cancer	rs4489787	1.00E-06
2012/3/31	Tao S	22219177	A genome-wide search for loci interacting with known prostate cancer risk-associated genetic variants.	Prostate cancer	rs1571801	6.00E-07
2012/3/31	Tao S	22219177	A genome-wide search for loci interacting with known prostate cancer risk-associated genetic variants.	Prostate cancer	rs8102476	7.00E-07
2012/3/31	Tao S	22219177	A genome-wide search for loci interacting with known prostate cancer risk-associated genetic variants.	Prostate cancer	rs4430796	1.00E-06
2012/3/31	Tao S	22219177	A genome-wide search for loci interacting with known prostate cancer risk-associated genetic variants.	Prostate cancer	rs1916284	1.00E-06
2012/3/31	Tao S	22219177	A genome-wide search for loci interacting with known prostate cancer risk-	Prostate cancer	rs9649213	1.00E-06

			associated genetic variants.			
2012/3/31	Tao S	22219177	A genome-wide search for loci interacting with known prostate cancer risk-associated genetic variants.	Prostate cancer	rs4962416	1.00E-06
2012/3/31	Tao S	22219177	A genome-wide search for loci interacting with known prostate cancer risk-associated genetic variants.	Prostate cancer	rs1243647	1.00E-06
2012/3/31	Tao S	22219177	A genome-wide search for loci interacting with known prostate cancer risk-associated genetic variants.	Prostate cancer	rs7679673	1.00E-06
2012/3/31	Tao S	22219177	A genome-wide search for loci interacting with known prostate cancer risk-associated genetic variants.	Prostate cancer	rs12682851	2.00E-06
2012/3/31	Tao S	22219177	A genome-wide search for loci interacting with known prostate cancer risk-associated genetic variants.	Prostate cancer	rs7127900	3.00E-06
2012/3/31	Tao S	22219177	A genome-wide search for loci interacting with known prostate cancer risk-associated genetic variants.	Prostate cancer	rs13264970	4.00E-06
2012/3/31	Tao S	22219177	A genome-wide search for loci interacting with known prostate cancer risk-associated genetic variants.	Prostate cancer	rs13258681	4.00E-06
2012/3/31	Tao S	22219177	A genome-wide search for loci interacting with known prostate cancer risk-associated genetic variants.	Prostate cancer	rs13192613	3.00E-06
2012/3/31	Tao S	22219177	A genome-wide search for loci interacting with known prostate cancer risk-associated genetic variants.	Prostate cancer	rs11649743	3.00E-06
2012/3/31	Tao S	22219177	A genome-wide search for loci interacting with known prostate cancer risk-associated genetic variants.	Prostate cancer	rs1859962	3.00E-06
2012/3/31	Tao S	22219177	A genome-wide search for loci interacting with known prostate cancer risk-associated genetic variants.	Prostate cancer	rs6089829	3.00E-06
2012/3/31	Tao S	22219177	A genome-wide search for loci interacting with known prostate cancer risk-associated genetic variants.	Prostate cancer	rs2735839	3.00E-06
2012/3/31	Tao S	22219177	A genome-wide search for loci interacting with known prostate cancer risk-associated genetic variants.	Prostate cancer	rs7789197	3.00E-06
2012/3/31	Tao S	22219177	A genome-wide search for loci interacting with known prostate cancer risk-associated genetic variants.	Prostate cancer	rs7717572	3.00E-06
2012/3/31	Tao S	22219177	A genome-wide search for loci interacting with known prostate cancer risk-associated genetic variants.	Prostate cancer	rs2660753	3.00E-06
2012/3/31	Tao S	22219177	A genome-wide search for loci interacting with known prostate cancer risk-associated genetic variants.	Prostate cancer	rs998124	5.00E-06
2012/3/31	Tao S	22219177	A genome-wide search for loci interacting with known prostate cancer risk-associated genetic variants.	Prostate cancer	rs3789080	4.00E-06
2012/3/31	Tao S	22219177	A genome-wide search for loci interacting with known prostate cancer risk-associated genetic variants.	Prostate cancer	rs11605083	4.00E-06
2012/3/31	Tao S	22219177	A genome-wide search for loci interacting with known prostate cancer risk-	Prostate cancer	rs731174	5.00E-06

			associated genetic variants.			
2012/3/31	Tao S	22219177	A genome-wide search for loci interacting with known prostate cancer risk-associated genetic variants.	Prostate cancer	rs8057939	5.00E-06
2012/3/31	Tao S	22219177	A genome-wide search for loci interacting with known prostate cancer risk-associated genetic variants.	Prostate cancer	rs17021918	5.00E-06
2012/3/31	Tao S	22219177	A genome-wide search for loci interacting with known prostate cancer risk-associated genetic variants.	Prostate cancer	rs10456809	5.00E-06
2012/3/31	Tao S	22219177	A genome-wide search for loci interacting with known prostate cancer risk-associated genetic variants.	Prostate cancer	rs10486567	5.00E-06
2012/3/31	Tao S	22219177	A genome-wide search for loci interacting with known prostate cancer risk-associated genetic variants.	Prostate cancer	rs9351730	5.00E-06
2012/3/31	Tao S	22219177	A genome-wide search for loci interacting with known prostate cancer risk-associated genetic variants.	Prostate cancer	rs9364554	5.00E-06
2012/3/31	Tao S	22219177	A genome-wide search for loci interacting with known prostate cancer risk-associated genetic variants.	Prostate cancer	rs1866967	5.00E-06
2012/3/31	Tao S	22219177	A genome-wide search for loci interacting with known prostate cancer risk-associated genetic variants.	Prostate cancer	rs13398206	4.00E-06
2012/3/31	Tao S	22219177	A genome-wide search for loci interacting with known prostate cancer risk-associated genetic variants.	Prostate cancer	rs12317459	4.00E-06
2012/3/31	Tao S	22219177	A genome-wide search for loci interacting with known prostate cancer risk-associated genetic variants.	Prostate cancer	rs10896449	4.00E-06
2012/3/31	Tao S	22219177	A genome-wide search for loci interacting with known prostate cancer risk-associated genetic variants.	Prostate cancer	rs16901979	4.00E-06
2012/3/31	Tao S	22219177	A genome-wide search for loci interacting with known prostate cancer risk-associated genetic variants.	Prostate cancer	rs10277209	4.00E-06
2012/3/31	Tao S	22219177	A genome-wide search for loci interacting with known prostate cancer risk-associated genetic variants.	Prostate cancer	rs6763848	4.00E-06
2012/3/31	Tao S	22219177	A genome-wide search for loci interacting with known prostate cancer risk-associated genetic variants.	Prostate cancer	rs9623117	4.00E-06
2012/3/31	Tao S	22219177	A genome-wide search for loci interacting with known prostate cancer risk-associated genetic variants.	Prostate cancer	rs1465618	4.00E-06
2012/3/31	Tao S	22219177	A genome-wide search for loci interacting with known prostate cancer risk-associated genetic variants.	Prostate cancer	rs543686	4.00E-06
2012/3/31	Tao S	22219177	A genome-wide search for loci interacting with known prostate cancer risk-associated genetic variants.	Prostate cancer	rs6005451	4.00E-06
2012/3/31	Tao S	22219177	A genome-wide search for loci interacting with known prostate cancer risk-associated genetic variants.	Prostate cancer	rs6983267	4.00E-06
2012/3/31	Tao S	22219177	A genome-wide search for loci interacting with known prostate cancer risk-	Prostate cancer	rs445114	4.00E-06

			associated genetic variants.			
2012/3/31	Tao S	22219177	A genome-wide search for loci interacting with known prostate cancer risk-associated genetic variants.	Prostate cancer	rs5751168	4.00E-06
2012/3/31	Tao S	22219177	A genome-wide search for loci interacting with known prostate cancer risk-associated genetic variants.	Prostate cancer	rs6507016	4.00E-06
2012/3/31	Tao S	22219177	A genome-wide search for loci interacting with known prostate cancer risk-associated genetic variants.	Prostate cancer	rs2523395	2.00E-06
2012/3/31	Tao S	22219177	A genome-wide search for loci interacting with known prostate cancer risk-associated genetic variants.	Prostate cancer	rs5945619	4.00E-06
2012/3/31	Tao S	22219177	A genome-wide search for loci interacting with known prostate cancer risk-associated genetic variants.	Prostate cancer	rs1512268	2.00E-06
2012/3/31	Tao S	22219177	A genome-wide search for loci interacting with known prostate cancer risk-associated genetic variants.	Prostate cancer	rs16944141	3.00E-06
2012/3/31	Tao S	22219177	A genome-wide search for loci interacting with known prostate cancer risk-associated genetic variants.	Prostate cancer	rs784411	1.00E-07
2012/3/31	Tao S	22219177	A genome-wide search for loci interacting with known prostate cancer risk-associated genetic variants.	Prostate cancer	rs12621278	3.00E-06
2012/1/11	Nam RK	22130093	New variants at 10q26 and 15q21 are associated with aggressive prostate cancer in a genome-wide association study from a prostate biopsy screening cohort.	Prostate cancer	rs4775302	4.00E-08
2012/1/11	Nam RK	22130093	New variants at 10q26 and 15q21 are associated with aggressive prostate cancer in a genome-wide association study from a prostate biopsy screening cohort.	Prostate cancer	rs11199874	3.00E-10
2011/11/24	Haiman CA	22037553	A common variant at the TERT-CLPTM1L locus is associated with estrogen receptor-negative breast cancer.	Breast cancer	rs10069690	1.00E-10
2011/11/9	Wu X	22010048	A genome-wide association study identifies a novel susceptibility locus for renal cell carcinoma on 12p11.23.	Renal cell carcinoma	rs25422	5.00E-06
2011/11/9	Wu X	22010048	A genome-wide association study identifies a novel susceptibility locus for renal cell carcinoma on 12p11.23.	Renal cell carcinoma	rs718314	9.00E-10
2011/11/9	Wu X	22010048	A genome-wide association study identifies a novel susceptibility locus for renal cell carcinoma on 12p11.23.	Renal cell carcinoma	rs4980785	2.00E-06
2011/12/10	Barrett JH	21983787	Genome-wide association study identifies three new melanoma susceptibility loci.	Melanoma	rs401681	3.00E-08
2011/12/10	Barrett JH	21983787	Genome-wide association study identifies three new melanoma susceptibility loci.	Melanoma	rs7023329	7.00E-09
2011/12/10	Barrett JH	21983787	Genome-wide association study identifies three new melanoma susceptibility loci.	Melanoma	rs1393350	2.00E-13
2011/12/10	Barrett JH	21983787	Genome-wide association study identifies three new melanoma susceptibility loci.	Melanoma	rs45430	3.00E-09
2011/12/10	Barrett JH	21983787	Genome-wide association study identifies three new melanoma susceptibility loci.	Melanoma	rs258322	3.00E-27
2011/12/10	Barrett JH	21983787	Genome-wide association study identifies three new melanoma susceptibility loci.	Melanoma	rs35390	2.00E-07
2011/12/10	Barrett JH	21983787	Genome-wide association study identifies three new melanoma susceptibility loci.	Melanoma	rs6001027	2.00E-06
2011/12/10	Barrett JH	21983787	Genome-wide association study identifies three new melanoma susceptibility loci.	Melanoma	rs13016963	9.00E-10
2011/12/10	Barrett JH	21983787	Genome-wide association study identifies three new melanoma susceptibility loci.	Melanoma	rs1801516	3.00E-09

2011/11/2	Macgregor S	21983785	Genome-wide association study identifies a new melanoma susceptibility locus at 1q21.3.	Melanoma	rs3219090	9.00E-08
2011/11/2	Macgregor S	21983785	Genome-wide association study identifies a new melanoma susceptibility locus at 1q21.3.	Melanoma	rs7412746	9.00E-11
2011/10/7	Amos CI	21926416	Genome-wide association study identifies novel loci predisposing to cutaneous melanoma.	Melanoma	rs1722784	2.00E-06
2011/8/15	Peters U	21761138	Meta-analysis of new genome-wide association studies of colorectal cancer risk.	Colorectal cancer	rs7315438	6.00E-06
2011/8/15	Peters U	21761138	Meta-analysis of new genome-wide association studies of colorectal cancer risk.	Colorectal cancer	rs16892766	4.00E-07
2011/8/15	Peters U	21761138	Meta-analysis of new genome-wide association studies of colorectal cancer risk.	Colorectal cancer	rs4779584	2.00E-08
2011/8/15	Peters U	21761138	Meta-analysis of new genome-wide association studies of colorectal cancer risk.	Colorectal cancer	rs3802842	4.00E-07
2011/8/15	Peters U	21761138	Meta-analysis of new genome-wide association studies of colorectal cancer risk.	Colorectal cancer	rs4939827	1.00E-07
2011/8/22	Kote-Jarai Z	21743467	Seven prostate cancer susceptibility loci identified by a multi-stage genome-wide association study.	Prostate cancer	rs5919432	1.00E-08
2011/8/22	Kote-Jarai Z	21743467	Seven prostate cancer susceptibility loci identified by a multi-stage genome-wide association study.	Prostate cancer	rs2121875	4.00E-08
2011/8/22	Kote-Jarai Z	21743467	Seven prostate cancer susceptibility loci identified by a multi-stage genome-wide association study.	Prostate cancer	rs130067	3.00E-08
2011/8/22	Kote-Jarai Z	21743467	Seven prostate cancer susceptibility loci identified by a multi-stage genome-wide association study.	Prostate cancer	rs10187424	3.00E-15
2011/8/22	Kote-Jarai Z	21743467	Seven prostate cancer susceptibility loci identified by a multi-stage genome-wide association study.	Prostate cancer	rs10875943	7.00E-12
2011/8/22	Kote-Jarai Z	21743467	Seven prostate cancer susceptibility loci identified by a multi-stage genome-wide association study.	Prostate cancer	rs6763931	2.00E-08
2011/8/22	Kote-Jarai Z	21743467	Seven prostate cancer susceptibility loci identified by a multi-stage genome-wide association study.	Prostate cancer	rs7584330	3.00E-09
2011/7/30	Schumacher FR	21743057	Genome-wide association study identifies new prostate cancer susceptibility loci.	Prostate cancer	rs902774	5.00E-09
2011/7/30	Schumacher FR	21743057	Genome-wide association study identifies new prostate cancer susceptibility loci.	Prostate cancer	rs7629490	1.00E-07
2011/7/30	Schumacher FR	21743057	Genome-wide association study identifies new prostate cancer susceptibility loci.	Prostate cancer	rs7130881	9.00E-09
2011/7/30	Schumacher FR	21743057	Genome-wide association study identifies new prostate cancer susceptibility loci.	Prostate cancer	rs7501939	2.00E-06
2011/7/30	Schumacher FR	21743057	Genome-wide association study identifies new prostate cancer susceptibility loci.	Prostate cancer	rs1859962	3.00E-11
2011/7/30	Schumacher FR	21743057	Genome-wide association study identifies new prostate cancer susceptibility loci.	Prostate cancer	rs1016343	4.00E-10
2011/7/30	Schumacher FR	21743057	Genome-wide association study identifies new prostate cancer susceptibility loci.	Prostate cancer	rs2292884	4.00E-08
2011/7/30	Schumacher FR	21743057	Genome-wide association study identifies new prostate cancer susceptibility loci.	Prostate cancer	rs13252298	4.00E-06
2011/7/30	Schumacher FR	21743057	Genome-wide association study identifies new prostate cancer susceptibility loci.	Prostate cancer	rs742134	6.00E-06
2011/7/30	Schumacher FR	21743057	Genome-wide association study identifies new prostate cancer susceptibility loci.	Prostate cancer	rs445114	5.00E-07
2011/7/30	Schumacher FR	21743057	Genome-wide association study identifies new prostate cancer susceptibility loci.	Prostate cancer	rs6983267	9.00E-06
2011/7/30	Schumacher FR	21743057	Genome-wide association study identifies new prostate cancer susceptibility loci.	Prostate cancer	rs4242384	3.00E-16

2011/7/30	Schumacher FR	21743057	Genome-wide association study identifies new prostate cancer susceptibility loci.	Prostate cancer	rs10993994	5.00E-06
2011/7/30	Schumacher FR	21743057	Genome-wide association study identifies new prostate cancer susceptibility loci.	Prostate cancer	rs651164	2.00E-10
2011/7/20	Teerlink C	21706340	A unique genome-wide association analysis in extended Utah high-risk pedigrees identifies a novel melanoma risk variant on chromosome arm 10q.	Melanoma	rs17119461	7.00E-12
2011/6/1	Sanson M	21531791	Chromosome 7p11.2 (EGFR) variation influences glioma risk.	Glioma	rs498872	5.00E-11
2011/6/1	Sanson M	21531791	Chromosome 7p11.2 (EGFR) variation influences glioma risk.	Glioma	rs2252586	8.00E-08
2011/6/1	Sanson M	21531791	Chromosome 7p11.2 (EGFR) variation influences glioma risk.	Glioma	rs2157719	5.00E-16
2011/6/1	Sanson M	21531791	Chromosome 7p11.2 (EGFR) variation influences glioma risk.	Glioma	rs4295627	5.00E-21
2011/6/1	Sanson M	21531791	Chromosome 7p11.2 (EGFR) variation influences glioma risk.	Glioma	rs11979158	7.00E-08
2011/5/24	Spurdle AB	21499250	Genome-wide association study identifies a common variant associated with risk of endometrial cancer.	Endometrial cancer	rs673604	6.00E-06
2011/5/24	Spurdle AB	21499250	Genome-wide association study identifies a common variant associated with risk of endometrial cancer.	Endometrial cancer	rs4430796	7.00E-10
2011/4/13	Sehrawat B	21424380	Potential novel candidate polymorphisms identified in genome-wide association study for breast cancer susceptibility.	Breast cancer	rs10411161	7.00E-06
2011/4/13	Sehrawat B	21424380	Potential novel candidate polymorphisms identified in genome-wide association study for breast cancer susceptibility.	Breast cancer	rs1092913	2.00E-06
2011/3/4	Fletcher O	21263130	Novel breast cancer susceptibility locus at 9q31.2: results of a genome-wide association study.	Breast cancer	rs10510102	2.00E-06
2011/3/4	Fletcher O	21263130	Novel breast cancer susceptibility locus at 9q31.2: results of a genome-wide association study.	Breast cancer	rs3112612	4.00E-10
2011/3/4	Fletcher O	21263130	Novel breast cancer susceptibility locus at 9q31.2: results of a genome-wide association study.	Breast cancer	rs1562430	3.00E-11
2011/3/4	Fletcher O	21263130	Novel breast cancer susceptibility locus at 9q31.2: results of a genome-wide association study.	Breast cancer	rs4973768	2.00E-08
2011/3/4	Fletcher O	21263130	Novel breast cancer susceptibility locus at 9q31.2: results of a genome-wide association study.	Breast cancer	rs13387042	2.00E-10
2011/3/4	Fletcher O	21263130	Novel breast cancer susceptibility locus at 9q31.2: results of a genome-wide association study.	Breast cancer	rs4415084	8.00E-11
2011/3/4	Fletcher O	21263130	Novel breast cancer susceptibility locus at 9q31.2: results of a genome-wide association study.	Breast cancer	rs1219648	1.00E-30
2011/3/4	Fletcher O	21263130	Novel breast cancer susceptibility locus at 9q31.2: results of a genome-wide association study.	Breast cancer	rs865686	2.00E-10
2011/3/4	Fletcher O	21263130	Novel breast cancer susceptibility locus at 9q31.2: results of a genome-wide association study.	Breast cancer	rs3734805	1.00E-07
2011/1/13	Gudmundsson J	21160077	Genetic correction of PSA values using sequence variants associated with PSA levels.	Prostate-specific antigen levels	rs266849	6.00E-10

2011/1/13	Gudmundsson J	21160077	Genetic correction of PSA values using sequence variants associated with PSA levels.	Prostate-specific antigen levels	rs11067228	2.00E-11
2011/1/13	Gudmundsson J	21160077	Genetic correction of PSA values using sequence variants associated with PSA levels.	Prostate-specific antigen levels	rs17632542	3.00E-46
2011/1/15	Purdue MP	21131975	Genome-wide association study of renal cell carcinoma identifies two susceptibility loci on 2p21 and 11q13.3.	Renal cell carcinoma	rs7579899	2.00E-09
2011/1/15	Purdue MP	21131975	Genome-wide association study of renal cell carcinoma identifies two susceptibility loci on 2p21 and 11q13.3.	Renal cell carcinoma	rs4765623	3.00E-08
2011/1/15	Purdue MP	21131975	Genome-wide association study of renal cell carcinoma identifies two susceptibility loci on 2p21 and 11q13.3.	Renal cell carcinoma	rs7105934	8.00E-14
2010/12/9	Gaudet MM	21060860	Common genetic variants and modification of penetrance of BRCA2-associated breast cancer.	Breast cancer	rs2981575	1.00E-08
2010/12/9	Houlston RS	20972440	Meta-analysis of three genome-wide association studies identifies susceptibility loci for colorectal cancer at 1q41, 3q26.2, 12q13.13 and 20q13.33.	Colorectal cancer	rs4925386	2.00E-10
2010/12/9	Houlston RS	20972440	Meta-analysis of three genome-wide association studies identifies susceptibility loci for colorectal cancer at 1q41, 3q26.2, 12q13.13 and 20q13.33.	Colorectal cancer	rs11169552	2.00E-10
2010/12/9	Houlston RS	20972440	Meta-analysis of three genome-wide association studies identifies susceptibility loci for colorectal cancer at 1q41, 3q26.2, 12q13.13 and 20q13.33.	Colorectal cancer	rs10936599	3.00E-08
2010/12/9	Houlston RS	20972440	Meta-analysis of three genome-wide association studies identifies susceptibility loci for colorectal cancer at 1q41, 3q26.2, 12q13.13 and 20q13.33.	Colorectal cancer	rs6687758	2.00E-09
2010/12/9	Houlston RS	20972440	Meta-analysis of three genome-wide association studies identifies susceptibility loci for colorectal cancer at 1q41, 3q26.2, 12q13.13 and 20q13.33.	Colorectal cancer	rs6691170	1.00E-09
2010/12/8	Rothman N	20972438	A multi-stage genome-wide association study of bladder cancer identifies multiple susceptibility loci.	Bladder cancer	rs1014971	8.00E-12
2010/12/8	Rothman N	20972438	A multi-stage genome-wide association study of bladder cancer identifies multiple susceptibility loci.	Bladder cancer	rs11892031	1.00E-07
2010/12/8	Rothman N	20972438	A multi-stage genome-wide association study of bladder cancer identifies multiple susceptibility loci.	Bladder cancer	rs2294008	4.00E-11
2010/12/8	Rothman N	20972438	A multi-stage genome-wide association study of bladder cancer identifies multiple susceptibility loci.	Bladder cancer	rs710521	2.00E-10
2010/12/8	Rothman N	20972438	A multi-stage genome-wide association study of bladder cancer identifies multiple susceptibility loci.	Bladder cancer	rs8102137	2.00E-11
2010/12/8	Rothman N	20972438	A multi-stage genome-wide association study of bladder cancer identifies multiple susceptibility loci.	Bladder cancer	rs9642880	2.00E-18
2010/12/8	Rothman N	20972438	A multi-stage genome-wide association study of bladder cancer identifies multiple susceptibility loci.	Bladder cancer	rs1495741	4.00E-11
2010/12/8	Rothman N	20972438	A multi-stage genome-wide association study of bladder cancer identifies multiple susceptibility loci.	Bladder cancer	rs798766	4.00E-13

2010/10/15	Li J	20872241	A combined analysis of genome-wide association studies in breast cancer.	Breast cancer	rs7716600	7.00E-07
2010/10/15	Li J	20872241	A combined analysis of genome-wide association studies in breast cancer.	Breast cancer	rs3803662	4.00E-07
2010/10/7	Antoniou AC	20852631	A locus on 19p13 modifies risk of breast cancer in BRCA1 mutation carriers and is associated with hormone receptor-negative breast cancer in the general population.	Breast cancer	rs8170	2.00E-09
2010/10/7	Bolton KL	20852633	Common variants at 19p13 are associated with susceptibility to ovarian cancer.	Ovarian cancer	rs2363956	1.00E-07
2010/10/12	Goode EL	20852632	A genome-wide association study identifies susceptibility loci for ovarian cancer at 2q31 and 8q24.	Ovarian cancer	rs2072590	5.00E-14
2010/10/12	Goode EL	20852632	A genome-wide association study identifies susceptibility loci for ovarian cancer at 2q31 and 8q24.	Ovarian cancer	rs10088218	3.00E-09
2010/10/12	Goode EL	20852632	A genome-wide association study identifies susceptibility loci for ovarian cancer at 2q31 and 8q24.	Ovarian cancer	rs2084881	2.00E-06
2010/10/12	Goode EL	20852632	A genome-wide association study identifies susceptibility loci for ovarian cancer at 2q31 and 8q24.	Ovarian cancer	rs7521902	5.00E-06
2010/10/12	Goode EL	20852632	A genome-wide association study identifies susceptibility loci for ovarian cancer at 2q31 and 8q24.	Ovarian cancer	rs2665390	3.00E-07
2010/7/12	Turnbull C	20543847	Variants near DMRT1, TERT and ATF7IP are associated with testicular germ cell cancer.	Testicular germ cell cancer	rs3782181	2.00E-26
2010/7/12	Turnbull C	20543847	Variants near DMRT1, TERT and ATF7IP are associated with testicular germ cell cancer.	Testicular germ cell cancer	rs4624820	1.00E-14
2010/7/12	Turnbull C	20543847	Variants near DMRT1, TERT and ATF7IP are associated with testicular germ cell cancer.	Testicular germ cell cancer	rs2900333	6.00E-10
2010/7/12	Turnbull C	20543847	Variants near DMRT1, TERT and ATF7IP are associated with testicular germ cell cancer.	Testicular germ cell cancer	rs4635969	1.00E-23
2010/7/12	Turnbull C	20543847	Variants near DMRT1, TERT and ATF7IP are associated with testicular germ cell cancer.	Testicular germ cell cancer	rs755383	1.00E-23
2010/6/2	Turnbull C	20453838	Genome-wide association study identifies five new breast cancer susceptibility loci.	Breast cancer	rs614367	3.00E-15
2010/6/2	Turnbull C	20453838	Genome-wide association study identifies five new breast cancer susceptibility loci.	Breast cancer	rs704010	4.00E-09
2010/6/2	Turnbull C	20453838	Genome-wide association study identifies five new breast cancer susceptibility loci.	Breast cancer	rs2380205	5.00E-07
2010/6/2	Turnbull C	20453838	Genome-wide association study identifies five new breast cancer susceptibility loci.	Breast cancer	rs3757318	3.00E-06
2010/6/2	Turnbull C	20453838	Genome-wide association study identifies five new breast cancer susceptibility loci.	Breast cancer	rs909116	7.00E-07
2010/6/2	Turnbull C	20453838	Genome-wide association study identifies five new breast cancer susceptibility loci.	Breast cancer	rs10995190	5.00E-15

2010/6/2	Turnbull C	20453838	Genome-wide association study identifies five new breast cancer susceptibility loci.	Breast cancer	rs2981579	4.00E-31
2010/6/2	Turnbull C	20453838	Genome-wide association study identifies five new breast cancer susceptibility loci.	Breast cancer	rs1011970	3.00E-08
2010/3/29	Li Y	20304703	Genetic variants and risk of lung cancer in never smokers: a genome-wide association study.	Lung cancer	rs2352028	6.00E-06
2010/2/28	Petersen GM	20101243	A genome-wide association study identifies pancreatic cancer susceptibility loci on chromosomes 13q22.1, 1q32.1 and 5p15.33.	Pancreatic cancer	rs9543325	3.00E-11
2010/2/28	Petersen GM	20101243	A genome-wide association study identifies pancreatic cancer susceptibility loci on chromosomes 13q22.1, 1q32.1 and 5p15.33.	Pancreatic cancer	rs3790844	2.00E-10
2009/12/29	Eeles RA	19767753	Identification of seven new prostate cancer susceptibility loci through a genome-wide association study.	Prostate cancer	rs5759167	6.00E-29
2009/12/29	Eeles RA	19767753	Identification of seven new prostate cancer susceptibility loci through a genome-wide association study.	Prostate cancer	rs3123078	1.00E-19
2009/12/29	Eeles RA	19767753	Identification of seven new prostate cancer susceptibility loci through a genome-wide association study.	Prostate cancer	rs7127900	3.00E-33
2009/12/29	Eeles RA	19767753	Identification of seven new prostate cancer susceptibility loci through a genome-wide association study.	Prostate cancer	rs6465657	2.00E-08
2009/12/29	Eeles RA	19767753	Identification of seven new prostate cancer susceptibility loci through a genome-wide association study.	Prostate cancer	rs6545977	5.00E-07
2009/12/29	Eeles RA	19767753	Identification of seven new prostate cancer susceptibility loci through a genome-wide association study.	Prostate cancer	rs12500426	1.00E-11
2009/12/29	Eeles RA	19767753	Identification of seven new prostate cancer susceptibility loci through a genome-wide association study.	Prostate cancer	rs1327301	2.00E-10
2009/12/29	Eeles RA	19767753	Identification of seven new prostate cancer susceptibility loci through a genome-wide association study.	Prostate cancer	rs1512268	3.00E-30
2009/12/29	Eeles RA	19767753	Identification of seven new prostate cancer susceptibility loci through a genome-wide association study.	Prostate cancer	rs7679673	3.00E-14
2009/12/29	Eeles RA	19767753	Identification of seven new prostate cancer susceptibility loci through a genome-wide association study.	Prostate cancer	rs17021918	4.00E-15
2009/12/29	Eeles RA	19767753	Identification of seven new prostate cancer susceptibility loci through a genome-wide association study.	Prostate cancer	rs1465618	2.00E-08
2009/12/29	Eeles RA	19767753	Identification of seven new prostate cancer susceptibility loci through a genome-wide association study.	Prostate cancer	rs17181170	3.00E-08
2009/12/29	Eeles RA	19767753	Identification of seven new prostate cancer susceptibility loci through a genome-wide association study.	Prostate cancer	rs12621278	9.00E-23
2009/12/29	Eeles RA	19767753	Identification of seven new prostate cancer susceptibility loci through a genome-wide association study.	Prostate cancer	rs12155172	9.00E-06

2009/10/5	Gudmundsson J	19767754	Genome-wide association and replication studies identify four variants associated with prostate cancer susceptibility.	Prostate cancer	rs16901979	3.00E-14
2009/10/5	Gudmundsson J	19767754	Genome-wide association and replication studies identify four variants associated with prostate cancer susceptibility.	Prostate cancer	rs8102476	2.00E-11
2009/10/5	Gudmundsson J	19767754	Genome-wide association and replication studies identify four variants associated with prostate cancer susceptibility.	Prostate cancer	rs10934853	3.00E-10
2009/10/5	Gudmundsson J	19767754	Genome-wide association and replication studies identify four variants associated with prostate cancer susceptibility.	Prostate cancer	rs11228565	7.00E-12
2009/10/5	Gudmundsson J	19767754	Genome-wide association and replication studies identify four variants associated with prostate cancer susceptibility.	Prostate cancer	rs1447295	2.00E-19
2009/9/14	Broderick P	19654303	Deciphering the impact of common genetic variation on lung cancer risk: a genome-wide association study.	Lung cancer	rs1530057	3.00E-06
2009/9/14	Broderick P	19654303	Deciphering the impact of common genetic variation on lung cancer risk: a genome-wide association study.	Lung cancer	rs748404	1.00E-06
2009/9/14	Broderick P	19654303	Deciphering the impact of common genetic variation on lung cancer risk: a genome-wide association study.	Lung cancer	rs3117582	4.00E-10
2009/9/14	Broderick P	19654303	Deciphering the impact of common genetic variation on lung cancer risk: a genome-wide association study.	Lung cancer	rs8034191	3.00E-26
2009/9/14	Broderick P	19654303	Deciphering the impact of common genetic variation on lung cancer risk: a genome-wide association study.	Lung cancer	rs4975616	3.00E-09
2009/9/14	Broderick P	19654303	Deciphering the impact of common genetic variation on lung cancer risk: a genome-wide association study.	Lung cancer	rs4254535	5.00E-06
2009/9/14	Broderick P	19654303	Deciphering the impact of common genetic variation on lung cancer risk: a genome-wide association study.	Lung cancer	rs1926203	1.00E-06
2009/9/4	Amundadottir L	19648918	Genome-wide association study identifies variants in the ABO locus associated with susceptibility to pancreatic cancer.	Pancreatic cancer	rs505922	5.00E-08
2009/9/4	Song H	19648919	A genome-wide association study identifies a new ovarian cancer susceptibility locus on 9p22.2.	Ovarian cancer	rs3814113	5.00E-19
2009/7/16	Bishop DT	19578364	Genome-wide association study identifies three loci associated with melanoma risk.	Melanoma	rs2284063	2.00E-09
2009/7/16	Bishop DT	19578364	Genome-wide association study identifies three loci associated with melanoma risk.	Melanoma	rs4785763	6.00E-22
2009/7/12	Shete S	19578367	Genome-wide association study identifies five susceptibility loci for glioma.	Glioma	rs891835	8.00E-11
2009/7/12	Shete S	19578367	Genome-wide association study identifies five susceptibility loci for glioma.	Glioma	rs2853676	4.00E-14
2009/7/12	Wrensch M	19578366	Variants in the CDKN2B and RTEL1 regions are associated with high-grade glioma susceptibility.	Glioma (high-grade)	rs1412829	2.00E-10
2009/7/12	Wrensch M	19578366	Variants in the CDKN2B and RTEL1 regions are associated with high-grade glioma susceptibility.	Glioma (high-grade)	rs4809324	2.00E-09

2009/6/14	Rapley EA	19483681	A genome-wide association study of testicular germ cell tumor.	Testicular germ cell tumor	rs210138	1.00E-13
2009/6/14	Rapley EA	19483681	A genome-wide association study of testicular germ cell tumor.	Testicular germ cell tumor	rs4699052	2.00E-07
2009/6/14	Rapley EA	19483681	A genome-wide association study of testicular germ cell tumor.	Testicular germ cell tumor	rs995030	1.00E-31
2009/6/14	Rapley EA	19483681	A genome-wide association study of testicular germ cell tumor.	Testicular germ cell tumor	rs4657482	2.00E-06
2009/6/14	Rapley EA	19483681	A genome-wide association study of testicular germ cell tumor.	Testicular germ cell tumor	rs1508595	3.00E-30
2009/4/3	Thomas G	19330030	A multistage genome-wide association study in breast cancer identifies two new risk alleles at 1p11.2 and 14q24.1 (RAD51L1).	Breast cancer	rs16886165	5.00E-07
2009/4/3	Thomas G	19330030	A multistage genome-wide association study in breast cancer identifies two new risk alleles at 1p11.2 and 14q24.1 (RAD51L1).	Breast cancer	rs11249433	7.00E-10
2009/4/3	Thomas G	19330030	A multistage genome-wide association study in breast cancer identifies two new risk alleles at 1p11.2 and 14q24.1 (RAD51L1).	Breast cancer	rs999737	2.00E-07
2009/2/28	Gudmundsson J	19198613	Common variants on 9q22.33 and 14q13.3 predispose to thyroid cancer in European populations.	Thyroid cancer	rs944289	2.00E-09
2009/2/28	Gudmundsson J	19198613	Common variants on 9q22.33 and 14q13.3 predispose to thyroid cancer in European populations.	Thyroid cancer	rs965513	2.00E-27
2009/2/26	Sun J	19117981	Sequence variants at 22q13 are associated with prostate cancer risk.	Prostate cancer	rs9623117	5.00E-07
2008/12/1	Houlston RS	19011631	Meta-analysis of genome-wide association data identifies four new susceptibility loci for colorectal cancer.	Colorectal cancer	rs4444235	8.00E-10
2008/12/1	Houlston RS	19011631	Meta-analysis of genome-wide association data identifies four new susceptibility loci for colorectal cancer.	Colorectal cancer	rs10411210	5.00E-09
2008/12/1	Houlston RS	19011631	Meta-analysis of genome-wide association data identifies four new susceptibility loci for colorectal cancer.	Colorectal cancer	rs9929218	1.00E-08
2008/12/1	Houlston RS	19011631	Meta-analysis of genome-wide association data identifies four new susceptibility loci for colorectal cancer.	Colorectal cancer	rs961253	2.00E-10
2008/12/9	McKay JD	18978790	Lung cancer susceptibility locus at 5p15.33.	Lung cancer	rs1051730	1.00E-15
2008/11/25	Wang Y	18978787	Common 5p15.33 and 6p21.33 variants influence lung cancer risk.	Lung cancer	rs8042374	8.00E-12
2008/11/25	Brown KM	18488026	Common sequence variants on 20q11.22 confer melanoma susceptibility.	Melanoma	rs910873	1.00E-15
2008/11/25	Amos CI	18385676	Genome-wide association scan of tag SNPs identifies a susceptibility locus for lung cancer at 15q25.1.	Lung cancer	rs7626795	8.00E-06
2008/11/25	Amos CI	18385676	Genome-wide association scan of tag SNPs identifies a susceptibility locus for lung cancer at 15q25.1.	Lung cancer	rs2808630	7.00E-06
2008/11/25	Tenesa A	18372901	Genome-wide association scan identifies a colorectal cancer susceptibility locus on 11q23 and replicates risk loci at 8q24 and 18q21.	Colorectal cancer	rs7014346	9.00E-26

2008/11/25	Tomlinson IP	18372905	A genome-wide association study identifies colorectal cancer susceptibility loci on chromosomes 10p14 and 8q23.3.	Colorectal cancer	rs10795668	3.00E-13
2008/11/25	Gold B	18326623	Genome-wide association study provides evidence for a breast cancer risk locus at 6q22.33.	Breast cancer	rs2180341	3.00E-08
2008/11/25	Eeles RA	18264097	Multiple newly identified loci associated with prostate cancer susceptibility.	Prostate cancer	rs2660753	3.00E-08
2008/11/25	Eeles RA	18264097	Multiple newly identified loci associated with prostate cancer susceptibility.	Prostate cancer	rs9364554	6.00E-10
2008/11/25	Eeles RA	18264097	Multiple newly identified loci associated with prostate cancer susceptibility.	Prostate cancer	rs7931342	2.00E-12
2008/11/25	Eeles RA	18264097	Multiple newly identified loci associated with prostate cancer susceptibility.	Prostate cancer	rs2735839	2.00E-18
2008/11/25	Eeles RA	18264097	Multiple newly identified loci associated with prostate cancer susceptibility.	Prostate cancer	rs5945619	2.00E-09
2008/11/25	Gudmundsson J	18264098	Common sequence variants on 2p15 and Xp11.22 confer susceptibility to prostate cancer.	Prostate cancer	rs5945572	4.00E-13
2008/11/25	Gudmundsson J	18264098	Common sequence variants on 2p15 and Xp11.22 confer susceptibility to prostate cancer.	Prostate cancer	rs721048	8.00E-09
2008/11/25	Thomas G	18264096	Multiple loci identified in a genome-wide association study of prostate cancer.	Prostate cancer	rs4962416	2.00E-07
2008/11/25	Thomas G	18264096	Multiple loci identified in a genome-wide association study of prostate cancer.	Prostate cancer	rs10896449	2.00E-09
2008/11/25	Thomas G	18264096	Multiple loci identified in a genome-wide association study of prostate cancer.	Prostate cancer	rs10486567	2.00E-06
2008/11/25	Thomas G	18264096	Multiple loci identified in a genome-wide association study of prostate cancer.	Prostate cancer	rs4242382	3.00E-19
2008/11/25	Murabito JM	17903305	A genome-wide association study of breast and prostate cancer in the NHLBI's Framingham Heart Study.	Breast cancer	rs458685	6.00E-06
2008/11/25	Murabito JM	17903305	A genome-wide association study of breast and prostate cancer in the NHLBI's Framingham Heart Study.	Breast cancer	rs2075555	8.00E-08
2008/11/25	Murabito JM	17903305	A genome-wide association study of breast and prostate cancer in the NHLBI's Framingham Heart Study.	Breast cancer	rs1926657	2.00E-06
2008/11/25	Murabito JM	17903305	A genome-wide association study of breast and prostate cancer in the NHLBI's Framingham Heart Study.	Breast cancer	rs1154865	7.00E-07
2008/11/25	Murabito JM	17903305	A genome-wide association study of breast and prostate cancer in the NHLBI's Framingham Heart Study.	Breast cancer	rs10263639	3.00E-06
2008/11/25	Murabito JM	17903305	A genome-wide association study of breast and prostate cancer in the NHLBI's Framingham Heart Study.	Breast cancer	rs10490113	5.00E-06
2008/11/25	Murabito JM	17903305	A genome-wide association study of breast and prostate cancer in the NHLBI's Framingham Heart Study.	Breast cancer	rs1876206	6.00E-06
2008/11/25	Murabito JM	17903305	A genome-wide association study of breast and prostate cancer in the NHLBI's Framingham Heart Study.	Breast cancer	rs1978503	1.00E-06
2008/11/25	Murabito JM	17903305	A genome-wide association study of breast and prostate cancer in the NHLBI's Framingham Heart Study.	Breast cancer	rs6556756	5.00E-07
2008/11/25	Murabito JM	17903305	A genome-wide association study of breast and prostate cancer in the NHLBI's Framingham Heart Study.	Prostate cancer	rs4466137	3.00E-06
2008/11/25	Murabito JM	17903305	A genome-wide association study of breast and prostate cancer in the NHLBI's	Prostate cancer	rs10498792	3.00E-06

			Framingham Heart Study.			
2008/11/25	Tomlinson IP	18372905	A genome-wide association study identifies colorectal cancer susceptibility loci on chromosomes 10p14 and 8q23.3.	Colorectal cancer	rs16892766	3.00E-18
2008/11/25	Tomlinson IP	18372905	A genome-wide association study identifies colorectal cancer susceptibility loci on chromosomes 10p14 and 8q23.3.	Colorectal cancer	rs6983267	7.00E-11
2008/11/25	Tomlinson IP	18372905	A genome-wide association study identifies colorectal cancer susceptibility loci on chromosomes 10p14 and 8q23.3.	Colorectal cancer	rs10795668	3.00E-13
2008/11/25	Zanke BW	17618283	Genome-wide association scan identifies a colorectal cancer susceptibility locus on chromosome 8q24.	Colorectal cancer	rs10505477	3.00E-11
2008/11/25	Easton DF	17529967	Genome-wide association study identifies novel breast cancer susceptibility loci.	Breast cancer	rs2981582	2.00E-76
2008/11/25	Easton DF	17529967	Genome-wide association study identifies novel breast cancer susceptibility loci.	Breast cancer	rs3817198	3.00E-09
2008/11/25	Easton DF	17529967	Genome-wide association study identifies novel breast cancer susceptibility loci.	Breast cancer	rs981782	9.00E-06
2008/11/25	Stacey SN	17529974	Common variants on chromosomes 2q35 and 16q12 confer susceptibility to estrogen receptor-positive breast cancer.	Breast cancer	rs13387042	1.00E-13
2008/11/25	Stacey SN	17529974	Common variants on chromosomes 2q35 and 16q12 confer susceptibility to estrogen receptor-positive breast cancer.	Breast cancer	rs3803662	6.00E-19
2008/11/25	Gudmundsson J	17401366	Genome-wide association study identifies a second prostate cancer susceptibility variant at 8q24.	Prostate cancer	rs1447295	6.00E-18
2008/11/25	Gudmundsson J	17401366	Genome-wide association study identifies a second prostate cancer susceptibility variant at 8q24.	Prostate cancer	rs16901979	1.00E-12
2008/11/25	Yeager M	17401363	Genome-wide association study of prostate cancer identifies a second risk locus at 8q24.	Prostate cancer	rs1447295	2.00E-14
2008/11/25	Yeager M	17401363	Genome-wide association study of prostate cancer identifies a second risk locus at 8q24.	Prostate cancer	rs6983267	9.00E-13

Supplementary Table 4: Cancer-associated SNPs in Asian population extracted from GWAS Catalog

Date added to Catalog	First Author	PubMed ID	Study	Disease/trait	SNP ID	*p*-value
2012/10/23	Cheng I	22923026	Evaluating genetic risk for prostate cancer among Japanese and Latinos.	Prostate cancer	rs12653946	3.00E-07
2012/10/23	Cheng I	22923026	Evaluating genetic risk for prostate cancer among Japanese and Latinos.	Prostate cancer	rs1512268	5.00E-06
2012/10/23	Cheng I	22923026	Evaluating genetic risk for prostate cancer among Japanese and Latinos.	Prostate cancer	rs10505483	7.00E-15
2012/10/23	Cheng I	22923026	Evaluating genetic risk for prostate cancer among Japanese and Latinos.	Prostate cancer	rs17023900	6.00E-08
2012/10/23	Cheng I	22923026	Evaluating genetic risk for prostate cancer among Japanese and Latinos.	Prostate cancer	rs10503733	8.00E-08
2012/4/28	Kim HC	22452962	A genome-wide association study identifies a breast cancer risk variant in ERBB4 at 2q34: results from the Seoul Breast Cancer Study.	Breast cancer	rs13393577	9.00E-14
2012/3/17	Long J	22383897	Genome-wide association study in east Asians identifies novel susceptibility loci for breast cancer.	Breast cancer	rs9485372	4.00E-12
2012/3/17	Long J	22383897	Genome-wide association study in east Asians identifies novel susceptibility loci for breast cancer.	Breast cancer	rs9383951	2.00E-16
2012/3/17	Long J	22383897	Genome-wide association study in east Asians identifies novel susceptibility loci for breast cancer.	Breast cancer	rs7107217	5.00E-07
2012/3/8	Cha PC	22318345	A genome-wide association study identifies SNP in DCC is associated with gallbladder cancer in the Japanese population.	Gallbladder cancer	rs13294589	2.00E-06
2012/3/8	Cha PC	22318345	A genome-wide association study identifies SNP in DCC is associated with gallbladder cancer in the Japanese population.	Gallbladder cancer	rs975334	9.00E-07
2012/3/8	Cha PC	22318345	A genome-wide association study identifies SNP in DCC is associated with gallbladder cancer in the Japanese population.	Gallbladder cancer	rs10953615	9.00E-06
2012/3/8	Cha PC	22318345	A genome-wide association study identifies SNP in DCC is associated with gallbladder cancer in the Japanese population.	Gallbladder cancer	rs6869388	7.00E-06
2012/3/8	Cha PC	22318345	A genome-wide association study identifies SNP in DCC is associated with gallbladder cancer in the Japanese population.	Gallbladder cancer	rs7504990	7.00E-08
2012/1/18	Wu C	22158540	Genome-wide association study identifies five loci associated with susceptibility to pancreatic cancer in Chinese populations.	Pancreatic cancer	rs1547374	4.00E-13
2012/1/18	Wu C	22158540	Genome-wide association study identifies five loci associated with susceptibility to pancreatic cancer in Chinese populations.	Pancreatic cancer	rs2689154	6.00E-06
2012/1/18	Wu C	22158540	Genome-wide association study identifies five loci associated with susceptibility to pancreatic cancer in Chinese populations.	Pancreatic cancer	rs5768709	1.00E-10
2012/1/18	Wu C	22158540	Genome-wide association study identifies five loci associated with susceptibility to pancreatic cancer in Chinese populations.	Pancreatic cancer	rs372883	2.00E-13
2012/1/18	Wu C	22158540	Genome-wide association study identifies five loci associated with susceptibility to pancreatic cancer in Chinese populations.	Pancreatic cancer	rs4927850	2.00E-07

2012/1/18	Wu C	22158540	Genome-wide association study identifies five loci associated with susceptibility to pancreatic cancer in Chinese populations.	Pancreatic cancer	rs4269383	7.00E-07
2012/1/18	Wu C	22158540	Genome-wide association study identifies five loci associated with susceptibility to pancreatic cancer in Chinese populations.	Pancreatic cancer	rs10974531	5.00E-06
2012/1/18	Wu C	22158540	Genome-wide association study identifies five loci associated with susceptibility to pancreatic cancer in Chinese populations.	Pancreatic cancer	rs9363918	1.00E-06
2012/1/18	Wu C	22158540	Genome-wide association study identifies five loci associated with susceptibility to pancreatic cancer in Chinese populations.	Pancreatic cancer	rs9573163	5.00E-13
2012/1/18	Wu C	22158540	Genome-wide association study identifies five loci associated with susceptibility to pancreatic cancer in Chinese populations.	Pancreatic cancer	rs2255280	4.00E-10
2012/1/18	Wu C	22158540	Genome-wide association study identifies five loci associated with susceptibility to pancreatic cancer in Chinese populations.	Pancreatic cancer	rs12413624	5.00E-11
2011/12/2	Shi Y	22037551	A genome-wide association study identifies new susceptibility loci for non-cardia gastric cancer at 3q13.31 and 5p13.1.	Gastric cancer	rs13361707	8.00E-29
2011/12/2	Shi Y	22037551	A genome-wide association study identifies new susceptibility loci for non-cardia gastric cancer at 3q13.31 and 5p13.1.	Gastric cancer	rs9841504	2.00E-09
2011/9/30	Cai Q	21908515	Genome-wide association study identifies breast cancer risk variant at 10q21.2: results from the Asia Breast Cancer Consortium.	Breast cancer	rs10822013	6.00E-09
2011/9/30	Cai Q	21908515	Genome-wide association study identifies breast cancer risk variant at 10q21.2: results from the Asia Breast Cancer Consortium.	Breast cancer	rs2048672	6.00E-06
2011/9/27	Ahn MJ	21866343	The 18p11.22 locus is associated with never smoker non-small cell lung cancer susceptibility in Korean populations.	Non-small cell lung cancer	rs11080466	1.00E-06
2011/8/3	Hu Z	21725308	A genome-wide association study identifies two new lung cancer susceptibility loci at 13q12.12 and 22q12.2 in Han Chinese.	Lung cancer	rs2736100	1.00E-27
2011/8/3	Hu Z	21725308	A genome-wide association study identifies two new lung cancer susceptibility loci at 13q12.12 and 22q12.2 in Han Chinese.	Lung cancer	rs4488809	7.00E-26
2011/8/3	Hu Z	21725308	A genome-wide association study identifies two new lung cancer susceptibility loci at 13q12.12 and 22q12.2 in Han Chinese.	Lung cancer	rs36600	6.00E-13
2011/8/3	Hu Z	21725308	A genome-wide association study identifies two new lung cancer susceptibility loci at 13q12.12 and 22q12.2 in Han Chinese.	Lung cancer	rs753955	2.00E-12
2011/6/16	Wu C	21642993	Genome-wide association study identifies three new susceptibility loci for esophageal squamous-cell carcinoma in Chinese populations.	Esophageal cancer	rs2274223	4.00E-20
2011/6/16	Wu C	21642993	Genome-wide association study identifies three new susceptibility loci for esophageal squamous-cell carcinoma in Chinese populations.	Esophageal cancer	rs9868873	1.00E-07
2011/6/16	Wu C	21642993	Genome-wide association study identifies three new susceptibility loci for esophageal squamous-cell carcinoma in Chinese populations.	Esophageal cancer	rs10058728	5.00E-09
2011/6/16	Wu C	21642993	Genome-wide association study identifies three new susceptibility loci for esophageal squamous-cell carcinoma in Chinese populations.	Esophageal cancer	rs2014300	8.00E-22

2011/2/25	Cui R	21242260	Common variant in 6q26-q27 is associated with distal colon cancer in an Asian population.	Colorectal cancer	rs7758229	8.00E-09
2011/2/25	Cui R	21242260	Common variant in 6q26-q27 is associated with distal colon cancer in an Asian population.	Colorectal cancer	rs6983267	2.00E-08
2010/12/9	Wu C	21118971	Genome-wide interrogation identifies YAP1 variants associated with survival of small-cell lung cancer patients.	Small-cell lung cancer	rs716274	9.00E-08
2010/12/9	Sato Y	21079520	Genome-wide association study on overall survival of advanced non-small cell lung cancer patients treated with carboplatin and paclitaxel.	Non-small cell lung cancer	rs9981861	4.00E-06
2010/12/9	Sato Y	21079520	Genome-wide association study on overall survival of advanced non-small cell lung cancer patients treated with carboplatin and paclitaxel.	Non-small cell lung cancer	rs1209950	3.00E-07
2010/12/9	Sato Y	21079520	Genome-wide association study on overall survival of advanced non-small cell lung cancer patients treated with carboplatin and paclitaxel.	Non-small cell lung cancer	rs1656402	8.00E-08
2010/10/15	Yoon KA	20876614	A genome-wide association study reveals susceptibility variants for non-small cell lung cancer in the Korean population.	Non-small cell lung cancer	rs16951095	7.00E-06
2010/10/15	Yoon KA	20876614	A genome-wide association study reveals susceptibility variants for non-small cell lung cancer in the Korean population.	Non-small cell lung cancer	rs2131877	2.00E-08
2010/10/15	Yoon KA	20876614	A genome-wide association study reveals susceptibility variants for non-small cell lung cancer in the Korean population.	Non-small cell lung cancer	rs9303196	8.00E-06
2010/9/23	Abnet CC	20729852	A shared susceptibility locus in PLCE1 at 10q23 for gastric adenocarcinoma and esophageal squamous cell carcinoma.	Esophageal cancer and gastric cancer	rs3765524	2.00E-09
2010/9/23	Abnet CC	20729852	A shared susceptibility locus in PLCE1 at 10q23 for gastric adenocarcinoma and esophageal squamous cell carcinoma.	Esophageal cancer and gastric cancer	rs3781264	4.00E-09
2010/9/23	Abnet CC	20729852	A shared susceptibility locus in PLCE1 at 10q23 for gastric adenocarcinoma and esophageal squamous cell carcinoma.	Esophageal cancer and gastric cancer	rs738722	1.00E-08
2010/9/23	Abnet CC	20729852	A shared susceptibility locus in PLCE1 at 10q23 for gastric adenocarcinoma and esophageal squamous cell carcinoma.	Esophageal cancer and gastric cancer	rs4072037	4.00E-07
2010/9/11	Takata R	20676098	Genome-wide association study identifies five new susceptibility loci for prostate cancer in the Japanese population.	Prostate cancer	rs1983891	8.00E-08
2010/9/11	Takata R	20676098	Genome-wide association study identifies five new susceptibility loci for prostate cancer in the Japanese population.	Prostate cancer	rs7501939	1.00E-12
2010/9/11	Takata R	20676098	Genome-wide association study identifies five new susceptibility loci for prostate cancer in the Japanese population.	Prostate cancer	rs7837688	1.00E-25
2010/9/11	Takata R	20676098	Genome-wide association study identifies five new susceptibility loci for prostate cancer in the Japanese population.	Prostate cancer	rs1456315	2.00E-29
2010/9/11	Takata R	20676098	Genome-wide association study identifies five new susceptibility loci for prostate cancer in the Japanese population.	Prostate cancer	rs339331	2.00E-12
2010/9/11	Takata R	20676098	Genome-wide association study identifies five new susceptibility loci for prostate cancer in the Japanese population.	Prostate cancer	rs9284813	5.00E-09

2010/9/11	Takata R	20676098	Genome-wide association study identifies five new susceptibility loci for prostate cancer in the Japanese population.	Prostate cancer	rs13385191	8.00E-08
2010/9/11	Takata R	20676098	Genome-wide association study identifies five new susceptibility loci for prostate cancer in the Japanese population.	Prostate cancer	rs10993994	3.00E-08
2010/9/11	Takata R	20676098	Genome-wide association study identifies five new susceptibility loci for prostate cancer in the Japanese population.	Prostate cancer	rs9600079	3.00E-09
2010/9/10	Low SK	20686608	Genome-wide association study of pancreatic cancer in Japanese population.	Pancreatic cancer	rs6464375	4.00E-07
2010/9/10	Low SK	20686608	Genome-wide association study of pancreatic cancer in Japanese population.	Pancreatic cancer	rs9502893	3.00E-07
2010/9/10	Low SK	20686608	Genome-wide association study of pancreatic cancer in Japanese population.	Pancreatic cancer	rs10088262	4.00E-06
2010/9/10	Low SK	20686608	Genome-wide association study of pancreatic cancer in Japanese population.	Pancreatic cancer	rs6879627	8.00E-06
2010/9/10	Low SK	20686608	Genome-wide association study of pancreatic cancer in Japanese population.	Pancreatic cancer	rs708224	3.00E-07
2010/9/10	Low SK	20686608	Genome-wide association study of pancreatic cancer in Japanese population.	Pancreatic cancer	rs225190	6.00E-06
2010/9/10	Low SK	20686608	Genome-wide association study of pancreatic cancer in Japanese population.	Pancreatic cancer	rs3016539	7.00E-06
2010/9/10	Low SK	20686608	Genome-wide association study of pancreatic cancer in Japanese population.	Pancreatic cancer	rs6711606	4.00E-06
2010/9/10	Low SK	20686608	Genome-wide association study of pancreatic cancer in Japanese population.	Pancreatic cancer	rs2257205	8.00E-06
2010/9/10	Low SK	20686608	Genome-wide association study of pancreatic cancer in Japanese population.	Pancreatic cancer	rs1427593	7.00E-06
2010/9/10	Low SK	20686608	Genome-wide association study of pancreatic cancer in Japanese population.	Pancreatic cancer	rs12615966	7.00E-06
2010/9/10	Low SK	20686608	Genome-wide association study of pancreatic cancer in Japanese population.	Pancreatic cancer	rs1886449	9.00E-06
2010/9/10	Low SK	20686608	Genome-wide association study of pancreatic cancer in Japanese population.	Pancreatic cancer	rs2039553	7.00E-06
2010/9/10	Low SK	20686608	Genome-wide association study of pancreatic cancer in Japanese population.	Pancreatic cancer	rs4924935	8.00E-06
2010/9/10	Low SK	20686608	Genome-wide association study of pancreatic cancer in Japanese population.	Pancreatic cancer	rs7832232	5.00E-06
2010/9/10	Low SK	20686608	Genome-wide association study of pancreatic cancer in Japanese population.	Pancreatic cancer	rs1585440	9.00E-06
2010/9/10	Low SK	20686608	Genome-wide association study of pancreatic cancer in Japanese population.	Pancreatic cancer	rs6736997	6.00E-06
2010/7/12	Long J	20585626	Identification of a functional genetic variant at 16q12.1 for breast cancer risk: results from the Asia Breast Cancer Consortium.	Breast cancer	rs4784227	1.00E-28
2009/2/28	Zheng W	19219042	Genome-wide association study identifies a new breast cancer susceptibility locus at 6q25.1.	Breast cancer	rs2046210	2.00E-15
